# Reference intervals reimagined with IRIS for earlier detection and better disease monitoring

**DOI:** 10.1038/s41598-026-52500-z

**Published:** 2026-05-22

**Authors:** Murih Pusparum, Wendy P. J. den Elzen, Olivier Thas, Gökhan Ertaylan

**Affiliations:** 1 Environmental Intelligence, Flemish Institute for Technological Research (VITO), IndustriezoneVlasmeer 5, 2400 Mol, Belgium; 2 Data Science Institute, I-Biostat, Hasselt University, Hasselt, 3500 Belgium; 3 Laboratory Specialized Diagnostics & Research, Department of Laboratory Medicine, Amsterdam UMC, University of Amsterdam, 1105 AZ Amsterdam, The Netherlands; 4 Amsterdam Public Health Research Institute & Amsterdam Gastroenterology Endocrinology Metabolism, 1105 AZ Amsterdam, The Netherlands; 5 Department of Mathematics, Computer Science and Statistics, Ghent University, 9000 Ghent, Belgium; 6 National Institute for Applied Statistics Research Australia (NIASRA), University of Wollongong, Wollongong, NSW 2500 Australia

**Keywords:** Reference interval, Chronic disease, Proteomics, Metabolomics, Clinical tests, Precision medicine, Biomarkers, Computational biology and bioinformatics, Diseases, Medical research

## Abstract

Reference intervals (RIs) are the universal established methodology for interpreting numerical clinical data by comparing individual test results to population-based benchmarks. The quality of these RIs significantly influences individual-level decision-making. This study aims to answer the question on how we can effectively leverage an individual’s personal data in conjunction with that of their peers to compute individual reference intervals (IRIs) for key clinical parameters relevant to disease detection and progression. We describe the IRIS workflow that includes prior data processing and data quality check procedures. The computation of IRI involves the test results of multiple “healthy” data points from the same subject(s) and also from the peers. The model adjusts for covariates like sex and age, enhancing accuracy. The workflow demonstrated the potential utility of IRI in clinical and omics data from two longitudinal studies. For healthy populations, IRIs showed diagnostic value in chronic diseases, while in diseased cohorts, they enabled effective disease monitoring. An integrated application IRIS has been developed, incorporating all described steps in an easy-to-use tool in research and/or clinical practice. The IRI may assist in (1) early detection of disease transition in chronic diseases and (2) monitoring personal disease progression. It facilitates the detection of small deviations in clinical measurements, either using standard clinical biochemistry test results or omics data. With adequate data infrastructure, the IRIS workflow can be integrated into clinical practice by embedding personalised biomarker baselines into AI-enabled decision support systems Such integration provides a complementary layer that enhances the sensitivity and specificity of clinical alerts and risk stratification. Ultimately, this approach has the potential to transform personalised disease diagnosis, management, and patient outcomes.

**Supplementary Information:**

The online version contains supplementary material available at 10.1038/s41598-026-52500-z.

## Introduction

Effective statistical methods for handling personal data in clinical settings remain a challenge, despite advancements in AI and big data analytics that improve patient care and early disease detection^1–4^. It is still common practice today when visiting a doctor, patients often undergo lab tests for disease biomarkers. Results within the reference interval and, if accompanied by favourable physical examinations or past results, are considered normal. If results fall outside this range, further testing may be recommended.

The current methods for estimating population reference intervals (PRIs) primarily rely on cross-sectional data, which involves studying a group of healthy individuals. In this context, “healthy” typically refers to individuals without known acute or chronic disease and not receiving treatments expected to affect the biomarker of interest, as defined by study-specific inclusion and exclusion criteria. Recently, new approaches have emerged that utilise indirect procedures, where PRIs are determined based on large datasets of routine clinical tests rather than directly sampling healthy individuals and calculating PRIs^5–8^. However, it is important to note that these PRIs obtained from such methods still possess a population interpretation. This constraint makes them difficult to be implemented for early disease diagnosis, since patients at the early stage often behave similarly as the general healthy population. The novel concept of Individual Reference Intervals (IRI) has been introduced; it uses an individual’s past test results (and peers) data and therefore, allows for a more accurate interpretation of (future) test results^9,10^. This is because the IRI is more specific to the individual and may be narrower than a PRI, making the IRI more powerful for assisting in the early diagnosis of disease.

In the last few years, extensive efforts have been made for integrating multi-omics data in order to provide a better understanding at the molecular level of diseases, including chronic diseases^11,12^. Unlike the clinical biochemistry data, omics technologies usually give vast information in a high-throughput and multidimensional fashion. It is therefore considered cost effective and comprehensive^13^. Some omics technologies, such as proteomics and metabolomics, are now even frequently incorporated in routine biological and medical studies^14^. Since most of the omics data types are relatively new and not being routinely implemented in clinical practice, there are no established reference intervals for these datatypes.

By definition, chronic diseases require medical attention that last for one year or more, and they may progress over time^15^. Patients with chronic diseases are frequently diagnosed late, when symptoms are set, and the prognosis is poor. From this perspective, utilising omics data in defining disease sub-types and relationships between chronic diseases is self-evident, the IRI approach is instrumental in the diagnosis of disease onset.

In this manuscript, we aim to effectively leverage individuals personal and peer data to compute *personalised* reference intervals for a key parameter(s) that hold significance in the detection of disease onset and progression. An overview of this concept and examples of its implementations in a real-life clinical practice is presented on clinical biochemistry and omics data (metabolomics and proteomics). With both datasets we have performed an association analysis for discovering the relationships in several chronic diseases (CVD, chronic kidney diseases, and IBS). We employ the recent IRI estimation methodology and its extensions developed by Pusparum et al. for calculating all IRIs^10^, and provide an accompanying IRIS Shiny application that enables end-to-end processing and estimation of IRIs in a reproducible and user-friendly manner (*dsi-uhasselt.shinyapps.io/IRIS/*). In addition, we evaluate and compare the performance of two approaches for estimating IRIs, highlighting their respective strengths in longitudinal monitoring settings. We also show two crucial roles of IRI in early detection of disease transition and in monitoring personal disease progression.

## Methods

### Datasets

Three datasets are used: the IAM Frontier (IAF) data, the irritable bowel syndrome (IBS) data, and the MIMIC-III data for a small comparison study between different IRI methods^16–18^. The IAF dataset comes from a unique small-scale, high-dimensional longitudinal cohort study that ran for 12 months (13 time points) in 30 healthy subjects, consisting of 15 male and 15 female participants. Further details on the study design and data collection can be consulted in Supplementary Document Sect. 1. In this article we consider the clinical biochemistry, proteomics, and metabolomics features of the IAF study. The clinical biochemistry data consists of 88 clinical features that were monthly measured by an accredited clinical laboratory. Furthermore, bi-monthly data of 249 metabolites measured by NMR metabolomics analytical techniques and 266 proteins from OLINK cardiovascular and inflammation panels were included in our analyses^19–21^. All subjects participated in the study have given their consent for displaying their anonymised pseudo-names for research purposes.

The IBS data were collected from a longitudinal case-control study that observed and compared healthy subjects to two types of IBS patients: IBS-C (constipation-predominant) and IBS-D (diarrhoea predominant)^17^. In this study, a total of 77 participants were asked to donate their stool samples on a monthly basis until the sixth month. However, not all of them provided full samples; therefore, we only include participants with at least three-time points measurements. Monthly metagenomic sequencing and NMR metabolomics were also assessed, together with a dietary survey of food intake and symptom severity at each visit. For this article, the longitudinal NMR metabolomics dataset consisting of 24 metabolites are analysed. Table 1 presents a further description of both datasets.

**Table 1:** Overview of the two datasets used in this article (IAF and IBS). ^*†*^Free of chronic diseases at baseline.

Cohort	Male (*n*)	Female (*n*)	Age (median)	Number of features
IAF-Healthy^*†*^	15	15	45–59 (50.5)	88 clinical, 249﻿ metabolites, 266﻿ proteins
IBS-Healthy^*†*^	5	17	23–59 (32)	24 metabolites
IBS-C	1	18	20–63 (43)	
IBS-D	7	16	21–61 (35)	

In the collection and use of the IAF and the IBS datasets, several steps have been taken to ensure compliance with ethical guidelines and to safeguard individual privacy. Informed consents were obtained from all participants, ensuring they were fully aware of how their data would be used. Additionally, robust data security measures, including encryption and anonymisation were implemented to protect sensitive information and prevent any unauthorised access or identification of individuals. The IAF study was approved by the ethics committee of the Antwerp University Hospital (*RegN°: B300201938600*).

The MIMIC-III data comes from a large, de-identified collection of intensive care unit (ICU) records containing detailed, time-stamped clinical measurements^18^. In this study, serum creatinine was selected as a representative laboratory biomarker because it is routinely measured in ICU patients, has sufficient longitudinal coverage, and reflects changes in kidney function that often occur when blood circulation to organs becomes compromised during critical illness. Clinical deterioration was defined by the initiation of vasopressor therapy, a clinician-driven escalation of care used to support blood pressure and maintain adequate blood flow to vital organs when a patient can no longer do so on their own. Vasopressor initiation provides a precisely time-stamped and clinically meaningful event that is largely independent of creatinine measurements. All laboratory values were aligned relative to the time of vasopressor initiation and partitioned into four non-overlapping windows: a baseline window (− 72 to − 24 h) used to estimate individual reference intervals, a buffer window (− 24 to − 12 h) excluded to avoid contamination by early physiological changes, a prediction window (− 12 to 0 h) used to evaluate early-warning performance, and a post-event window (0 to + 24 h) used for confirmatory analyses only. Details of data extraction, cohort selection, and preprocessing steps are provided in the Supplementary Document Sect. 7). Access to the MIMIC-III database was granted through *PhysioNet* following completion of required data-use training, and the use of this fully de-identified dataset is approved under a waiver of informed consent by the institutional review boards of the contributing institutions.

For a real-world IRI implementation, the IRIS *Shinyapp* can be utilised where users can upload their data and receive the results. Importantly, while data will be processed on the *Shinyapp* server, no data will be retained or stored on the server after the analysis. Nevertheless, we strongly recommend that users take proactive measures to anonymise subject identifiers prior to uploading, as part of best data protection practices. In real-world laboratory and hospital settings, the use of dedicated data storage solutions would be essential for the secure management of both input data and results. Such systems should comply with the General Data Protection Regulation (GDPR) to ensure the privacy and security of personal data are maintained at all stages of the process. A demonstration of the IRIS *Shinyapp*, including its user interface and practitioner-oriented workflow, is provided in the Supplementary Document Sect. 8.

### Overview of IRI methods

The Penalised Joint Quantile Model 2 (PJQM2) can be implemented for simultaneously estimating the lower and the upper bounds of the IRIs^10^. This method does not rely on distributional assumptions, but it makes use of quantile models that inherit the typical flexibility of the statistical models. The model also allows for covariate adjustment by simply including additional terms for any covariates. If, for example, age affects the outcome, then better IRIs can be constructed if information on subjects of the same age can be shared; this is approximately the result of these extended models. As the original theoretical IRI implementation does not allow for it, as part of this article, we extend the earlier PJQM2 implementation so that covariates can be included in practice.

In our examples, sex and age are included with the reasoning subjects’ genetic and biological make ups are generally different between males and females, and their clinical as well as molecular level measurements may change as part of the aging process. With *τ*_1_ (*τ*_1_ = 0.025) and *τ*_2_ (*τ*_2_ = 0.975) representing the probabilities corresponding to the upper and the lower bounds of the IRIs, the corresponding quantiles are modelled as:

$$\:{Q}_{i}\left({\tau\:}_{1}\right)={\beta\:}_{0}+{u}_{i}+{z}_{i}{\beta\:}_{1}+{\beta\:}_{3}age+{\beta\:}_{4}sex$$

$$\:{Q}_{i}\left({\tau\:}_{2}\right)={\beta\:}_{0}+{u}_{i}+{z}_{i}{\beta\:}_{2}+{\beta\:}_{3}age+{\beta\:}_{4}sex,$$

where *β*_0_ is the fixed intercept and *u*_*i*_, *z*_*i*_ are the subject-specific effects. Further, *β*_1_ and *β*_2_ are the parameters that allow for the subject-specific IRI widths, *β*_3_ and *β*_4_ are the parameters for the age and sex effects. The model contains two subject-specific effects, *u*_*i*_ and *z*_*i*_, that allow for between-subject variability. The parameter estimation procedure essentially remains similar as in Pusparum et al.^10^, except that now Linear Quantile Mixed Model (LQMM)^22,23^ are fitted with age and sex as fixed-effects covariates. Estimation of these covariate coefficients directly influences the subject-specific effects *u*_*i*_ and *z*_*i*_, and consequently affects both the location and width of the estimated IRI bounds. Two penalty terms are incorporated in the estimation of *u*_*i*_ and *z*_*i*_ to ensure parameter identifiability and to induce shrinkage, which leads to information sharing across subjects. This mechanism is particularly important in settings with limited repeated measurements per subject, as commonly observed in clinical laboratory data. The optimal penalty levels are selected via cross-validation to achieve the nominal coverage of the IRIs. All IRI modelling and estimation steps were implemented in *R* version 4.4.3, with the PJQM2 framework providing a set of dedicated functions that build upon established packages, including *quantreg* and *lqmm*.

Recently, another method was proposed for the calculation of a personalised version of reference intervals (PrRI)^24^. However, their technique relies on normal distribution. In real-life settings, it is often difficult to check a distributional assumption, particularly in rather short-time series such as in the IAF and IBS datasets. A previous study compared the PrRI method with the proposed PJQM2, showing that this method struggles to detect test results that are far below the main clusters (lower outliers) in datasets that deviate from normal distribution^10^. We show in detail another comparison study of IRI in PrRI in two datasets in the Result section in this manuscript.

A recent framework was introduced to compute IRI using a parametric empirical Bayes approach^25^. In this framework, the IRI is obtained by estimating an individual’s latent homeostatic set point as a shrinkage estimate. The shrinkage involves a weighted average of the person’s observed mean and the population mean, where the weights depend on within- and between-subject biological variation. Conceptually, the method frames personalisation as a prediction problem under a hierarchical normal model, with key assumptions including approximate normality (often after transformation), stationary within-person variability, and a clear separation of analytical, within-subject, and between-subject variance components. Earlier, an adaptive reference interval was introduced^26^. This method generates reference intervals within a Bayesian framework, using an Expectation-Maximisation-based approximation. The individual-specific mean and individual-specific within-person variance are jointly estimated in a hierarchical model, allowing the interval to be updated sequentially as new data arrive. The approach assumes approximate distributional regularity (typically normality after transformation) and temporal stationarity within an individual, but relaxes the common assumption that all individuals share the same within-subject variance. Throughout this manuscript, the term *PrRI* refers to the method proposed by Coskun et al.^24^, while *IRI* denotes the individual reference interval estimated using the PJQM2 method^10^. The term *IRIS* (e.g., IRIS workflow, IRIS pipeline, IRIS feature selection) refers to the broader analytical framework for estimating IRI in high-dimensional data using PJQM2, including the pre-modelling steps such as target feature selection and quality control procedures to ensure stable longitudinal measurements within individuals.

### IRIS workflow

**Fig. 1:**
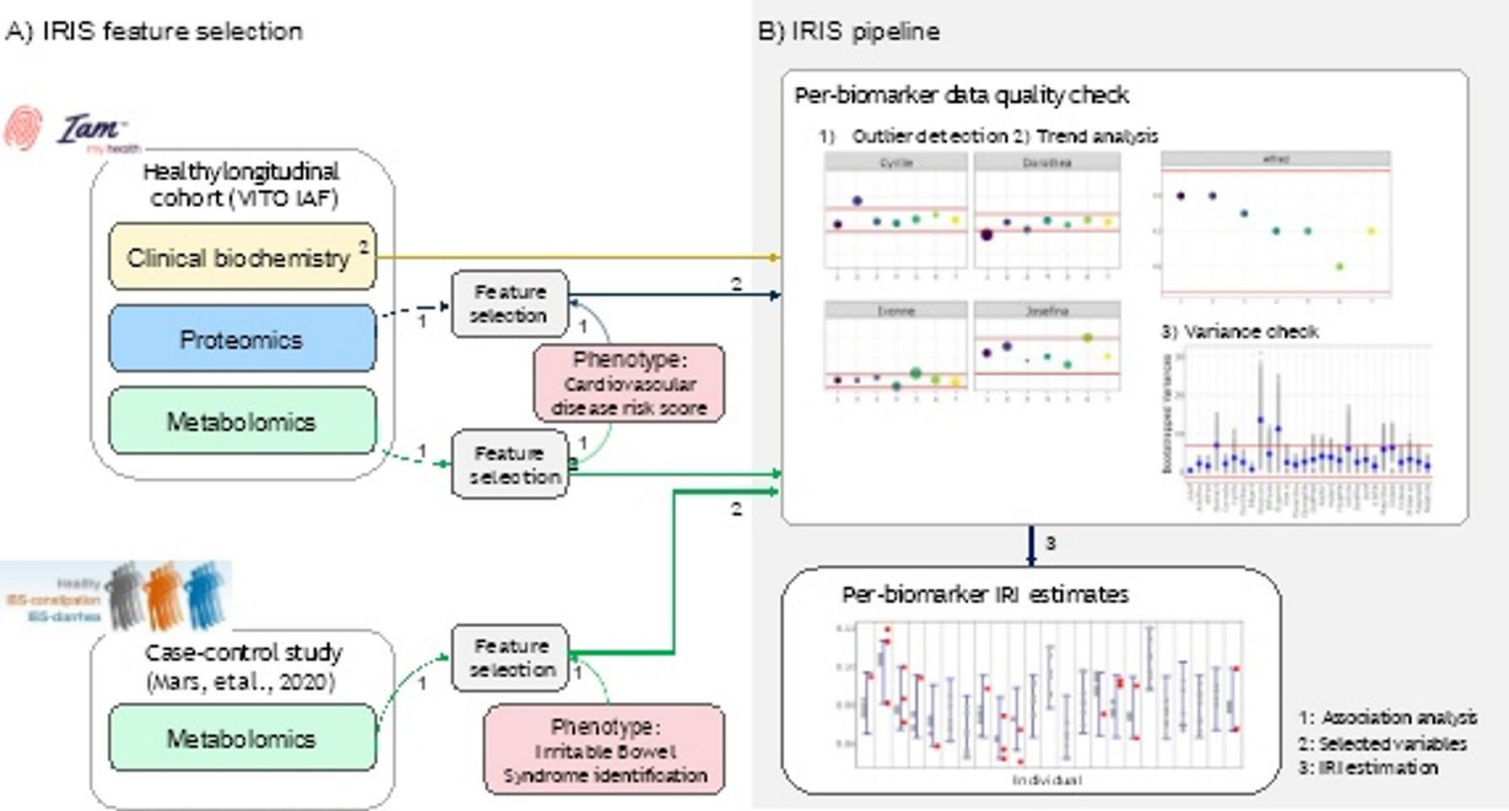
IRIS workflow for feature selection and IRI calculation. (**A**) Two clinical and omics datasets are used in this study (IAF and IBS). In the IRIS feature selection, association analyses are performed on each omics dataset, resulting in a set of potential biomarkers. (**B**) The selected biomarkers enter the IRIS pipeline; this includes a data quality check before the calculation of the IRI for each combination of a biomarker and a subject.

The IRIS workflow consists of two steps: IRIS feature selection and IRIS pipeline. In the first step as depicted in Fig. 1A, association analyses are performed between a set of features and the target disease phenotype of interest, and a few of the most relevant features are selected. For the implementation in preventive practice, we encourage to use phenotype in the form of predictive risk scores. When the diagnosis has been carried out referring to a particular chronic disease, the set of features may still be associated with the binary outcome resulted from the diagnosis (1 if disease is diagnosed and 0 otherwise). For each preventive and follow-up of diagnoses cases, the obtained IRIs would then respectively serve as, (1) a predictive tool to assist an early disease detection, and (2) as a tool for monitoring the disease progression. In this study, penalised mixed-effects models were applied to the IAF and IBS datasets as a feature-selection strategy for high-dimensional longitudinal data. This step is intended to be exploratory, providing candidate features to illustrate the IRIS workflow rather than to establish definitive disease biomarkers, particularly given the modest sample sizes. To mitigate potential overfitting, penalisation (LASSO/SCAD) and information criterion–based model selection (e.g., BIC) were used to promote model stability. This approach represents a well-established and robust framework that is particularly suitable for variable selection in omics-scale settings with repeated measurements. Further details of the modelling can be found in Supplementary Document Sect. 3.

In the second step, the selected features, we further refer them as *biomarkers*, are entered the IRIS pipeline. In this pipeline, we analyse each biomarker following the assumption that the subject observations per time series should be in a stable state. Outlier detection was performed using a robust median absolute deviation–based (MAD) approach with a conservative threshold. A stable state of observations per time series is assessed using the nonparametric Mann-Kendall test of monotonic trends^27^. Still in the pipeline, we finally compute the IRI for each biomarker. The detailed procedure is presented in Fig. 1B and justifications for the thresholds are provided in the Supplementary Document Sect. 2.

This IRIS feature selection step is intended to be performed by researchers, particularly when biomarkers of the target disease have not been discovered yet. It is also more plausible that researchers have access to study data with multi-omics or clinical biochemistry features. On the other hand, the IRIS pipeline has been fully automated and is therefore feasible for implementation in real-life clinical settings, including clinical laboratories and healthcare facilities (GPs). For omics-scale applications, we recommend addressing major technical covariates, such as batch or platform effects, during preprocessing prior to applying the IRIS workflow. In the datasets analysed in this study, technical corrections were performed upstream of feature selection and longitudinal modelling. In the last step of IRIS pipeline described above, the final IRI estimation therefore included only biological or clinical covariates, allowing the model to focus on meaningful biological variability rather than technical noise.

## Results

### Overview of the IRI journey

IRIs are developed for utilising individual’s as well as his/her peers’ historical data to gain meaningful insights for an early detection of disease onsets. One example of an IRI journey begins when a subject comes to general practitioners (GPs) to have a routine (medical) check-up. Often, when a subject has a check-up, biological samples such as blood and urine samples are collected, and laboratory test analyses are performed. The IRI can be calculated based on the results of these laboratory test analyses; the medical doctor might conclude that this subject is in a healthy condition, or they possess a risk in developing a disease. In the former case, we follow the general concept of estimating IRIs in healthy individuals. Otherwise, the IRIs can still be calculated where they would serve as a tool to personally assist and monitor the disease progression, see Fig. 4 for both cases.

At healthcare facilities such as GPs, the patients’ IRI in general would be implemented in the same way as the conventional population-based reference intervals; the IRI will be printed out in the document or digitally shared to the medical doctors and patients, showing the blood test results so the GPs can immediately compare them. IRIs will not be applicable for person with non-chronic or acute diseases, as the progressions often occur very fast and historical data in a stable condition are more difficult to obtain. The disease symptoms are also typically distinct and can be easily observed, thus IRIs could only provide little benefits.

### IRI and PrRI comparison

**Fig. 2:**
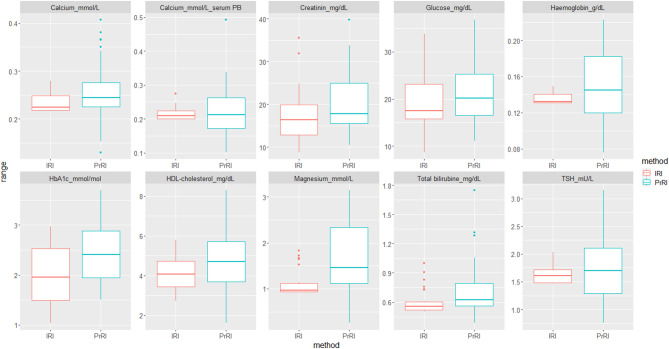
Distribution of the interval widths of IRI and PrRI in ten clinical biochemistry test results.

We implemented the IRI and PrRI methods to ten clinical biochemistry test results of the IAF dataset. A data quality check is performed before calculating the individual reference intervals; checking for trends in each time series and identifying any outlying observations. For each individual, we computed their interval estimates and the interval lengths (the difference between the upper and lower bounds), or we later call it the interval width. Figure 2 shows the distribution of interval widths for each test result across the two methods. Notably, the median width is generally lower in the IRI method, which also exhibits less variability. This outcome can be attributed to an assumption built into the IRI algorithm: although the reference intervals are personalised, both the location and width of these intervals are constrained by the overall population variance. This assumption is reasonable, as the individuals in the dataset are likely from the same population and share similar biological characteristics. Quantitatively, we found that PrRI tends to produce wider interval widths—16% and 15% larger based on the mean and median, respectively (see Supplementary Document Table S4). This suggests that, for some individuals, small deviations in test results may go unnoticed and be incorrectly classified as ‘normal.’ However, this hypothesis requires further validation, particularly with long-term follow-up data that include periods of health decline in the same subjects.

**Fig. 3:**
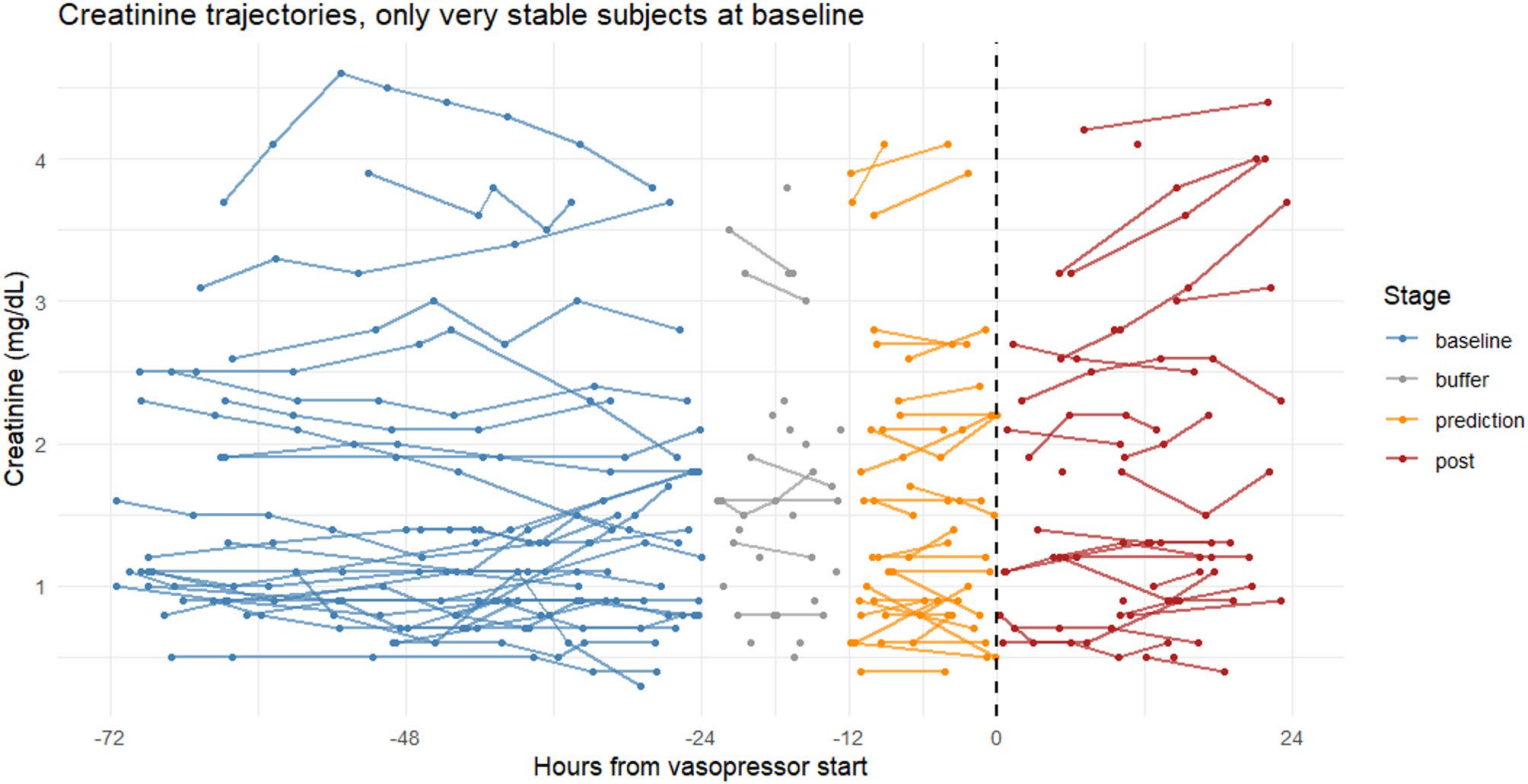
Individual trajectories of creatinine in 30 ICU stays. The measurements in the baseline window are used for estimating both IRI and PrRI, and those in the prediction and post window are used for computing the performance measures.

We further estimated IRI and PrRI for serum creatinine using the MIMIC-III dataset. After preprocessing, including the requirement of at least five creatinine measurements in the baseline window, 52 ICU stays (individuals) were retained for analysis. A subsequent data-quality step was applied to identify trajectories suitable for interval estimation, resulting in 30 eligible ICU stays with no significant monotonic trends and with similar within-subject variances. This subset provided both a stable period, represented by the baseline window, and a clinically deteriorating phase, represented by the post-vasopressor window, enabling method validation beyond what was possible in the IAF dataset. In addition, a buffer window and a prediction window were defined to separate baseline estimation from early-warning evaluation; the buffer window was used to exclude potentially transitional observations, while the prediction window was used to assess whether health deterioration could be detected earlier by IRI or PrRI. Individual creatinine trajectories across the four windows are shown in Fig. 3, and the comparative performance of IRI and PrRI, estimated using the baseline window and evaluated in the prediction window, is summarised in Table 2.

As summarised in Table 2, both IRI and PrRI demonstrated the ability to detect creatinine deviations prior to vasopressor initiation, with comparable median lead times (7 h for IRI vs. 8 h for PrRI) and patient-level sensitivities (0.47 vs. 0.43). Lead time is defined as the interval between the first detected deviation and the initiation of vasopressor therapy. However, important differences emerged in their statistical and clinical behaviour. The AUROC for PrRI could not be computed because, for some ICU stays, all baseline observations were identical, causing the estimated lower and upper bounds to collapse to a single value and yielding zero-width intervals with undefined normalised deviation scores. In contrast, IRI mitigates this issue by borrowing information from population-level variability. In contrast, IRI achieved a higher AUROC (0.38), indicating that it provides a continuous deviation score that offers some ability to distinguish between stable measurements and those obtained in the period preceding clinical deterioration. A perfect specificity, as observed for PrRI, does not necessarily indicate superior performance. By design, reference intervals are constructed to achieve a nominal coverage, here set to 95%, within the data used for their estimation. This implies that approximately 95% of baseline observations should fall within the interval regardless of the method. For this reason, baseline specificity is primarily a calibration property rather than a discriminative performance metric, particularly when computed on the same data used for interval estimation. In contrast, sensitivity is evaluated using independent prediction-window data that represents a period preceding clinical deterioration period and therefore provides a more meaningful measure of a method’s ability to detect clinically relevant deviations. The detailed description of these six performance metrics is available in the Supplementary Document Sect. 7.

**Table 2:** Six performance measures of IRI and PrRI.

Method	Specificity	Median lead time	Mean lead time	Sensitivity	AUROC	Post-breach rate
IRI	0.91	7.0 h	6.6 h	0.47	0.38	0.57
PrRI	1.00	8.0 h	6.9 h	0.43	NaN	0.60

### IRI for assisting diagnosis in clinical laboratory test

We first aim for estimating IRIs for some clinical biochemistry features available the IAF dataset. We estimated IRIs of creatinine; it is widely known that high levels of creatinine in the blood correspond to an acute kidney injury which could lead to chronic kidney diseases (CKD). We used the first seven time points of monthly clinical laboratory test data in the IAF study and took the last measurement at time point 12 or 13 ($\:\pm\:$6 months after the last measurement) for demonstrating the interpretation of the IRIs.

**Fig. 4:**
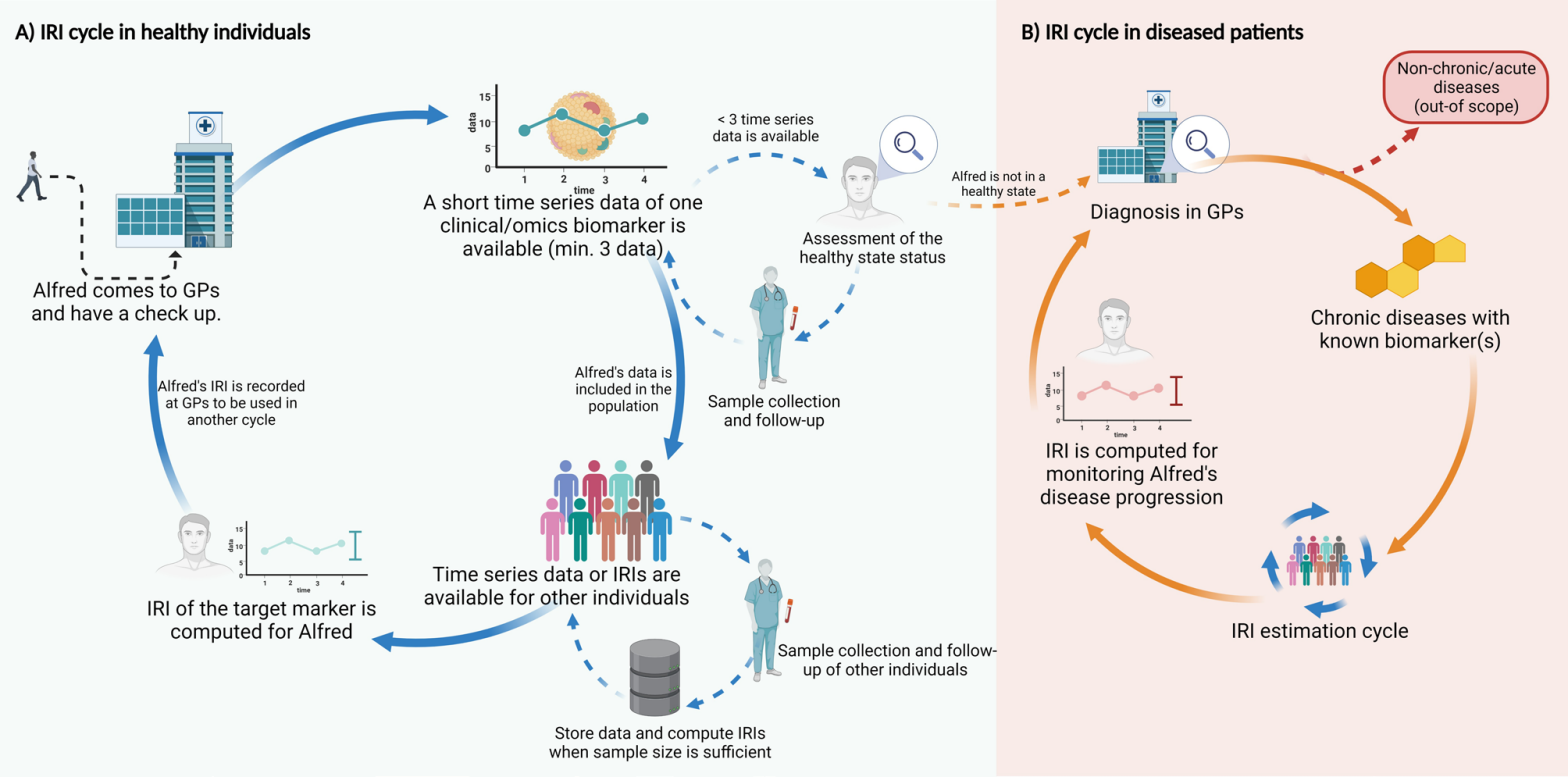
An illustration of estimating IRIs for a person, started from the visit to medical professionals. (**A**) The journey of the IRI cycle when the person is healthy, and (**B**) the alternative of IRI applications when the person is diagnosed with chronic diseases. At the end, the estimated IRIs will be recorded so it can be used for the next iteration both for the same person or for other individuals. IRIs will not be applicable for person with non-chronic or acute diseases, as the disease progressions often occur very fast and hence the patients are not in a stable state. Created with BioRender.com.

**Fig. 5:**
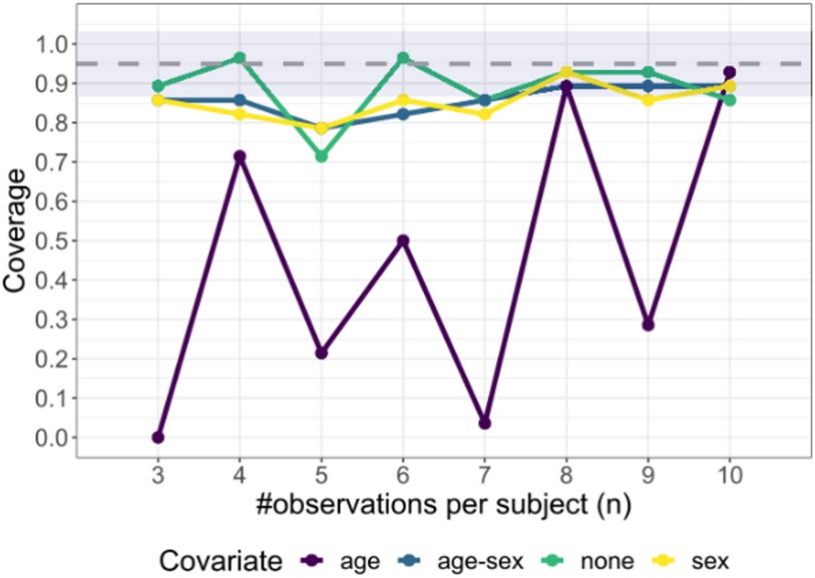
Coverage of IRIs in different model with covariates. Coverage (± std. deviation in the blue area) in IAF creatinine with different observations per time series fitted in different models. Model with only age as covariate gives unstable coverages. In the model with age and sex, only sex, and without covariates, at least seven observations per time series are needed to produce IRIs with coverage close to 95%. Small sample size data were used; hence a general recommendation cannot be drawn.

Before estimating the IRIs, as part of the IRIS pipeline, we have first done the data quality check. The trend analysis and the variance checking showed that one subject had an increasing monotonic trend, and two subjects had distinct variances as compared to their peers. Therefore, these three subjects were excluded from the IRI estimation. Results on this data quality step can be consulted in the Supplementary Document Figures S2–S4. We have also evaluated the IRI performance of this feature; we found that the IRI model should include sex as a covariate to produce good coverages, where at least seven observations per time series are needed, see Fig. 5. The Supplementary Document Sect. 4 explains the full procedure of this IRI performance evaluation.

**Fig. 6:**
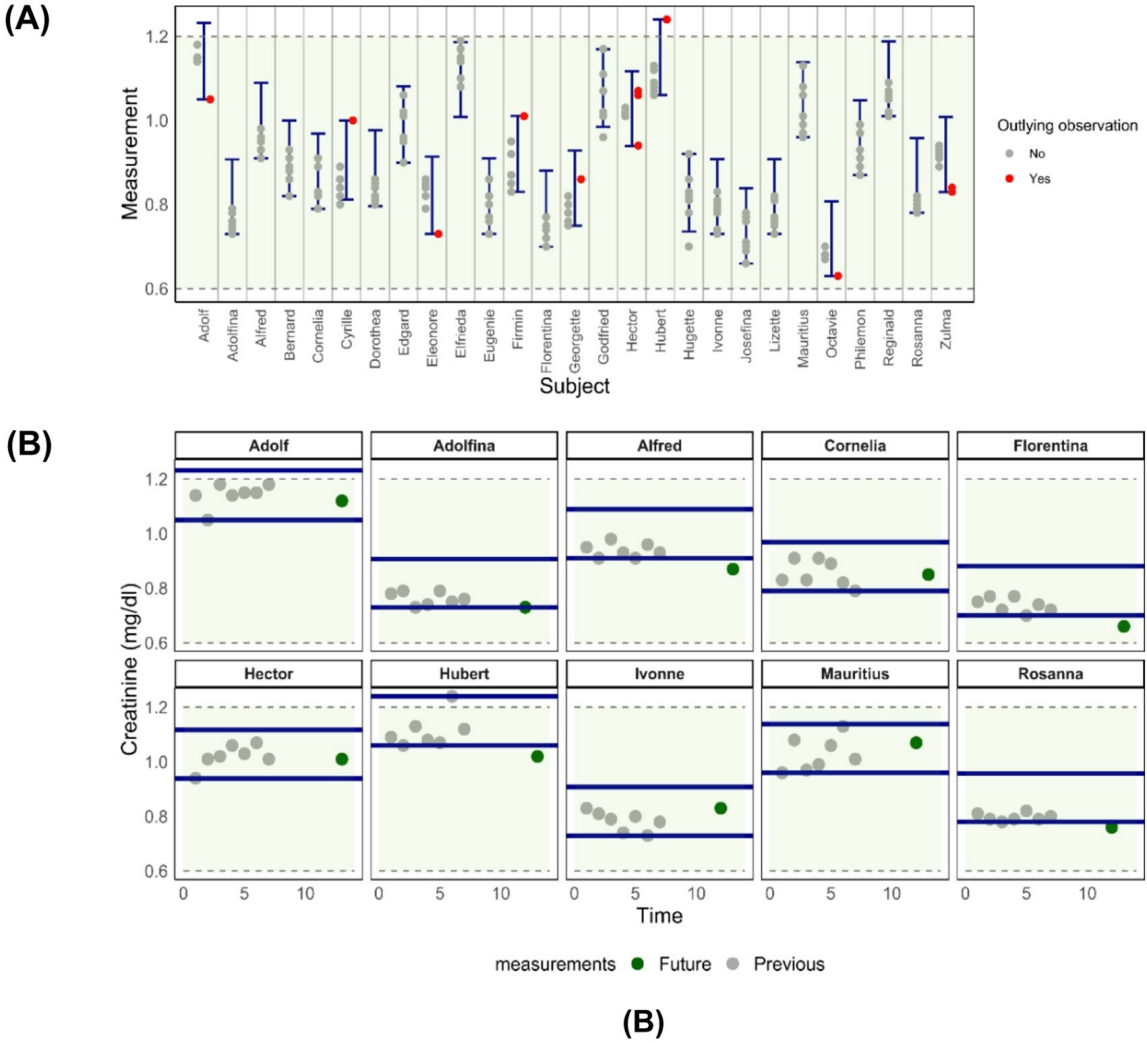
IRIs of creatinine in the IAF clinical biochemistry data. (**A**) The estimated IRIs of creatinine (mg/dl), with age and sex as additional covariates in the model. The grey circle dots refer to the subject observations used for the IRI estimation. Outlying observations, i.e., observations outside the MAD threshold of each subject time series, are included and they are flagged and showed as red dots. (**B**) The estimated IRIs of creatinine (mg/dl) of ten subjects in the IAF clinical biochemistry data. Seven previous measurements (grey circles) were used for the estimation. The blue horizontal lines represent the IRIs, the shaded green areas are the population-based reference intervals. A green dot is presented for each subject referring to a future measurement i.e., the last measurements collected in the study and not used for the IRI estimation. The IRIs should be used to interpret the future measurements. E.g., the new measurement of Hubert suggests a potential creatinine abnormality.

Figure 6A shows the IRI estimates of the creatinine level, computed using our extended model of PJQM2 method with age and sex as covariates. As expected, the IRIs are stretched around each subject observations and the locations as well as the widths vary between the subjects. We also see, for example, Adolfina’s upper bound is expanded farther away from her measurements. This is in fact a desirable consequence of the between-subject information-sharing property of the method^10^: Adolfina has overall smaller observations than the other subjects, and this is reflected in her IRI, which adapts to Adolfina’s own data, but also expands into the direction of the bulk of the data.

The use of the IRIs in practice is demonstrated in Fig. 6B, where the historical data (used for estimating the IRIs) and new measurements are plotted together with the IRIs. From the results we can see that the future measurements of some subjects are below the lower IRI bounds. These last measurements can be an early sign of hypocreatinemia resulting from the failures of liver function and low-protein diet. Had those new measurements been in upper borderline or outside the upper bound, it might have been an early caution of CKD onset. Note that we do not claim that a disease diagnosis can be made solely based on the use of IRIs. The IRIs may replace the population-based reference intervals and provide a personalised reading that should still be accompanied by other types of health examination and investigation of the current symptoms. Thus, the out-of-IRI measurements should not give an immediate decision on disease onset/progression, but rather flag them for a further examination.

### IRIs in omics for early CVD diagnosis based on CVD risk scores

Clinical laboratory tests are often considered as a gold standard in assisting disease diagnosis; around 70% of all clinical diagnoses are influenced by laboratory data [28]. Alternatively, we have also seen the prospective of omics data such as proteomics and metabolomics in supplementing medical diagnosis, as they are likely to be close to phenotype and therefore may benefit as a disease’s biomarker [11]. In this study, by using the IAF data we examined the most predictive metabolites and proteins with reference to cardiovascular diseases (CVD). In the same data, we discovered that the male participants have 1.1–35.4% risk of 10-year CVD event(s), whereas the risk is only 0.3–3.9% in females.

From the fitted models in the association analysis described in the previous section, we identified five and ten most discriminating metabolites and proteins related to the 10-year CVD risk scores. See Supplementary Document Sect. 3.3 for the detailed results of the feature selection and data quality check procedures. In this manuscript, for illustration purposes, we chose citrate from the metabolomics dataset as it gives the largest effect size.

**Fig. 7:**
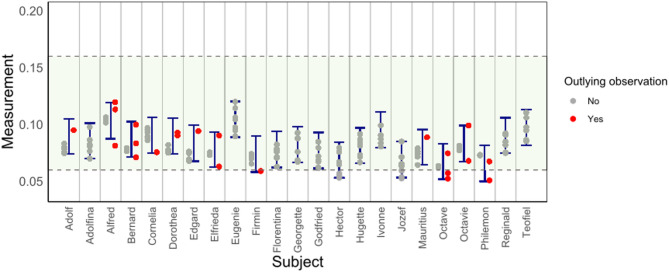
IRIs of citrate in the IAF metabolomics data. The estimated IRIs of citrate (in mmol/l) with age and sex as additional covariates in the model. The grey circle dots refer to the subject observations used in the estimation. Outlying observations, i.e., observations outside the MAD threshold of each subject time series, are included and they are flagged and shown as red dots.

Figure 7 shows the estimated IRIs of citrate, and similarly, these IRIs can be used for interpreting the future measurements of the subjects. A negative coefficient was estimated for citrate, meaning that higher citrate levels are more favourable with reference to CVD. The IRI interpretation may hence be more focused on the future measurements below the lower bound. Results of other selected metabolites and proteins can be found in the Supplementary Document Sect. 5.1.

### IRI as a monitoring tool for subjects with irritable bowel syndrome (IBS)

For the IBS dataset, we aim to estimate IRIs for biomarkers measured from both healthy and diseased individuals diagnosed with IBS. Unlike the routine reference intervals that provide normal values for detecting abnormalities, here we show how we can utilise IRIs for disease monitoring. The IRIS feature selection procedure was applied for the NMR metabolomics measurements in the IBS data. The fitted generalised linear mixed models with *ℓ*_1_ penalisation suggests the three most discriminating metabolites: hypoxanthine, glucose, and b-arabinose. In this manuscript, we show the analysis of hypoxanthine, as the largest effect size is contributed by this metabolite.

**Fig. 8:**
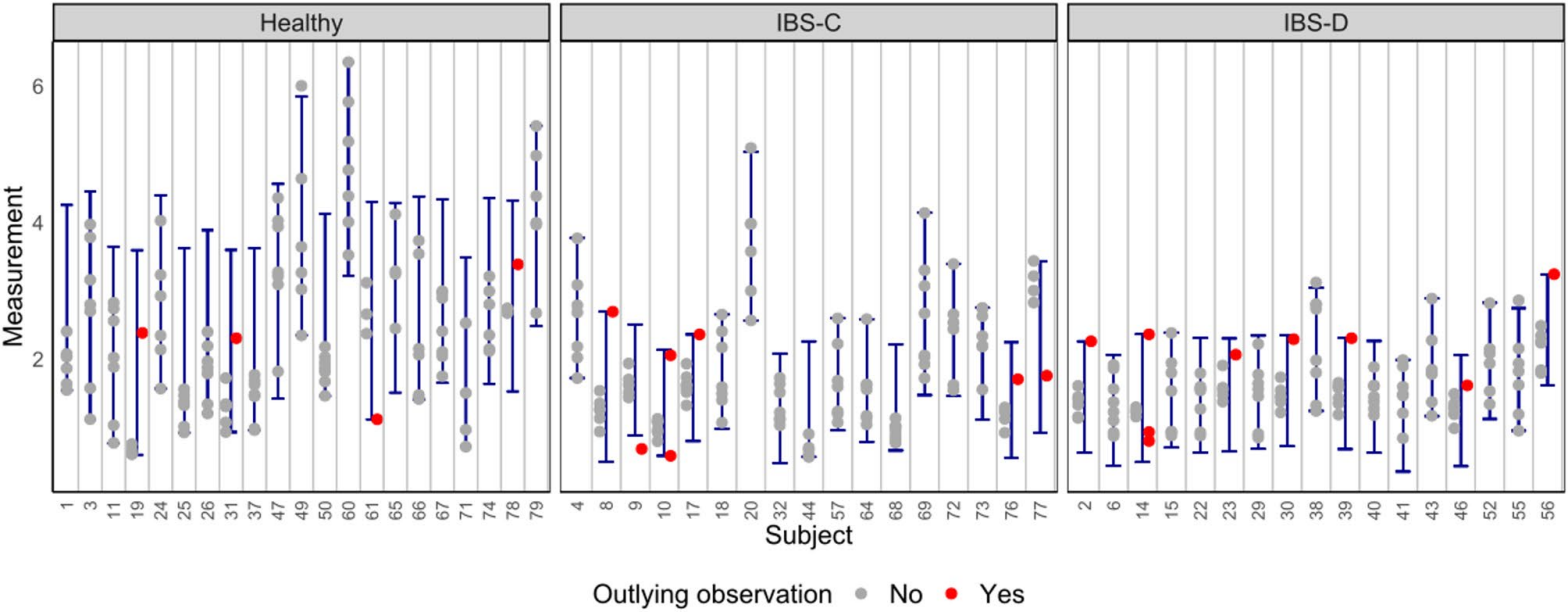
The estimated IRIs of hypoxanthine (relative abundance) in the IBS metabolomics data. IRIs for the healthy cohort, IBS-C and IBS-D patients.

Figure 8 presents the estimated IRIs for hypoxanthine for the three types of cohorts, after the data quality check was done in the IRIS pipeline. We clearly see that healthy subjects in general have larger hypoxanthine IRIs, both in terms of location as in terms of width, than the IBS patients. It has been shown that fecal hypoxanthine abundances were significantly lower in IBS-C and IBS-D patients, and therefore the decline of hypoxanthine abundances has implication to the IBS pathogenesis [17]. Similarly, we also observed that the IRI widths are clearly smaller in IBS-C and IBS-D patients, as compared to the healthy subjects (see Supplementary Document Table S3).

The healthy IRIs can be interpreted as the normal values, where if these subjects have new measurements outside their IRIs, especially below the lower bound, it should be an early warning of a potential IBS disease. The interpretation becomes slightly different for the IBS IRIs. For IBS patients, the estimated IRIs can serve as a monitoring tool for disease progression, either an improvement or a decline, because of for example, an intervention from drug therapy or medication. To illustrate, if the next hypoxanthine measurement of subject 2 in the IBS-D group is larger by one unit than their IRI, it then would suggest an improvement. Certainly, this analysis should also be still supported by professional assessments. Several IRIs from the IBS-C group appear to deviate from their peers, e.g. IRIs of subject 4, 20, and 69. These IRIs are similar in location and width as the healthy IRIs, and they perfectly capture the within-subject variation. If the individuals in this group are truly IBS-C patients, these IRIs demonstrate that the IBS state can differ from one subject to another with reference to hypoxanthine. Results of other selected metabolites can be consulted in the Supplementary Document Sect. 5.2.

## Discussion

In this study, we extend our earlier work on individual reference intervals (IRI) by introducing **IRIS**, a generalised and covariate-extended workflow for analysing clinical and omics data to support early diagnosis and longitudinal monitoring of chronic diseases. While previous work focused primarily on the methodological development of IRI estimation, the present study emphasises **practical implementation**, including integration of biological covariates, application to heterogeneous clinical and omics datasets, and deployment through an accessible *Shinyapp* (*dsi-uhasselt.shinyapps.io/IRIS/*). IRIs are personalised in that they account for patterns from both an individual’s own longitudinal data and information borrowed from peers within the population. As discussed below, the results should be interpreted as proof-of-concept evaluations given the modest cohort sizes.

For computing IRIs, the subject’s observations per time series should come from a population in a stable state. This corresponds to two possible scenarios: (1) if the IRIs are estimated from healthy individuals, the data needs to certainly come from a healthy population, and (2) if the IRIs are estimated for monitoring the chronic disease progression, the data should come from patients with chronic disease in their most stable state. An online *Shinyapp* IRIS application has been developed to operationalise the full IRIS workflow, allowing researcher and practitioners to apply IRI estimation in a reproducible and user-friendly manner. The IRIS workflow also incorporates a set of predefined thresholds for stability screening and data quality control, which yielded qualitatively consistent findings across the datasets analysed, suggesting robustness to reasonable variations in these choices.

The presented results are based on two datasets demonstrating the IRIS applications. The first dataset includes only healthy subjects, while the second includes both healthy subjects and individuals with chronic diseases. Unlike traditional reference interval studies, where population estimates are generalised, the results of this study should not be generalised to other cohorts or populations. The IRI estimates are specific to each participating individual, with potential confounding factors already accounted for. However, due to the small sample size, results such as those from the feature selection process in identifying biomarkers associated with CVD should be interpreted cautiously. Validation in larger and more representative cohorts with CVD is strongly recommended. While the longitudinal structure of the data partially mitigates small cohort sizes by leveraging within-individual information, the limited number of subjects (*n* = 30 for IAF; *n* = 77 for IBS) may still affect the generalisability of performance estimates and the robustness of feature selection results. To partially address this limitation, we additionally evaluated the approach using an independent MIMIC-III cohort with a larger sample size, providing complementary evidence for the practical applicability of IRI; nevertheless, further validation in larger, prospective, and more heterogeneous cohorts remains necessary.

In terms of evaluation, IRIs also differ from both traditional population reference intervals and alternative personalised approaches such as PrRI. In the MIMIC-III analysis, both IRI and PrRI were able to identify early deviations in creatinine prior to clinical deterioration. Quantitatively, both approaches showed comparable lead times (median 7.0 h vs. 8.0 h; mean 6.6 h vs. 6.9 h) and sensitivities (0.47 vs. 0.43), while IRI achieved a measurable AUROC (0.38) whereas PrRI did not yield a defined value. However, PrRI frequently produced collapsed, zero-width intervals when baseline measurements were identical, resulting in undefined deviation scores and preventing meaningful discrimination. By borrowing information from population-level variability, IRI is more robust and yields informative deviation signals even when within-individual variability is limited, supporting the practical advantages of IRI for longitudinal personalised monitoring. These findings should be interpreted as feasibility results based on retrospective data and a proxy definition of clinical deterioration, and therefore provide preliminary support for potential clinical utility.

Individual reference intervals are not intended to replace established clinical decision thresholds, but to complement them by identifying subject-specific deviations that may occur while measurements remain within conventional population reference ranges. Accordingly, an out-of-IRI measurement should be interpreted as an indication of atypical change for a given individual, prompting closer monitoring or further clinical evaluation rather than an immediate, predefined clinical action. For example, a biomarker value that remains within the conventional population reference interval but falls outside an individual’s IRI may indicate an early, subject-specific change, suggesting additional clinical attention or follow-up. Such signals are intended to support individualised assessment in the context of the patient’s longitudinal history and overall clinical profile.

In real-world practice, it is common for individuals not to consistently track or have access to certain omics measurements related to specific diseases. However, this is not a limitation of the proposed approach. The IRI estimation procedure does not require all variables related to the disease to be present for analysis. The method is designed to estimate IRIs of variables independently. That said, when comprehensive omics data is available, particularly in high-dimensional contexts, our IRIS workflow can be implemented even more effectively to model and identify potential disease biomarkers. Furthermore, the IRI estimation method remains applicable to biomarkers that are well-established and commonly measured in practice. The approach is also well-suited for handling missing individual data points.

The IRIS workflow can substantially enhance clinical decision-making by enabling personalised, high-resolution interpretation of test results. By anchoring assessments to individual baselines, it has the potential to support earlier identification of clinically meaningful deviations and more precise monitoring of disease progression. For example, recent advances in high-throughput plasma proteomics and AI enhanced our ability to assay and learn about a broad swath of our plasma proteins identifying human ageing process to organ specific changes in health and disease^29^. This personalised approach can be readily implemented in healthcare settings such as general practitioners’ (GPs) facilities, where the IRIs could be printed out on documents or digitally shared with both medical professionals and patients.

Future studies with real-life clinical and omics data with a larger number of subjects and different time series will provide a multitude of examples of the IRI implementation in practice. Such studies can also enable more contextual recommendations for different clinical and omics features, including the potential extension of the IRIS framework to account for longer-term temporal patterns, such as seasonal or other cyclical biological variations.

## Supplementary Information

Below is the link to the electronic supplementary material.

Supplementary Material 1.

## Data Availability

The IBS datasets analysed during the current study are available in the Mendeley Data repository (https:/data.mendeley.com/datasets/29n2z5r5ph/3). The MIMIC-III data analysed in this study are available via PhysioNet to qualified researchers upon completion of the required data-use training and approval process. Due to participants’ privacy, the IAF longitudinal data is available upon request to the I AM Frontier Project Data Access Committee for further research (DataAccess.IAF@vito.be ). The sensitive nature of the data does not allow it to be deposited into public repositories. We welcome collaboration with other research groups focused on personalised prevention, offering data sharing opportunities to advance research and improve healthcare outcomes. The preprint version of this study is available in the medrxiv server (https:/doi.org/10.1101/2022.07.18.22277788). Supplementary documents are available online.

## Code availability

The underlying codes for the IAF and IBS application studies are available on https://github.com/iris-statlab/applications.
