# Supplementary material for: Reference intervals reimagined with IRIS for earlier detection and better disease monitoring
Source: Sci Rep. 2026 May 22;16:23387. doi: 10.1038/s41598-026-52500-z (PMC13408599; doi:10.1038/s41598-026-52500-z)
Supplement: Supplementary file 1 — Supplementary Material 1. [file 41598_2026_52500_MOESM1_ESM.pdf]

Supplementary Material

Reference intervals reimagined with IRIS for earlier detection and better disease monitoring

Murih Pusparum[1,2], Wendy P.J. den Elzen[3,4], Olivier Thas[2,5,6], and Gökhan Ertaylan[1]

[1]*Environmental Intelligence, Flemish Institute for Technological Research (VITO), Mol 2400, Belgium*
[2]*Data Science Institute, I-Biostat, Hasselt University, Hasselt 3500, Belgium*
[3]*Laboratory Specialized Diagnostics Research, Department of Laboratory Medicine, Amsterdam UMC, University of Amsterdam, 1105 AZ, Amsterdam, the Netherlands*
[4]*Amsterdam Public Health Research Institute Amsterdam Gastroenterology Endocrinology Metabolism, 1105 AZ, Amsterdam, The Netherlands*
[5]*Department of Mathematics, Computer Science and Statistics, Ghent University, Ghent 9000, Belgium*
[6]*National Institute for Applied Statistics Research Australia (NIASRA), Wollongong 2500, NSW, Australia*

1 IAM Frontier data

The IAM Frontier (IAF) data consist of clinical biochemistry, physiological, and multi-omics measurements collected from samples of 30 'apparently' healthy individuals. The study specifically targeted healthy subjects within the age range of 45-59. The subjects were selected based on the inclusion criteria of not suffering from a chronic disease, diagnosed and currently followed-up by a medical specialist, including asthma, chronic bronchitis, chronic obstructive pulmonary disease, emphysema, myocardial infarction, coronary heart disease (angina pectoris), other serious heart diseases, stroke (cerebral haemorrhage, cerebral thrombosis), diabetes, cancer (malignant tumour, also including leukaemia and lymphoma). The age range was selected because the highest prevalence of onset of these chronic diseases occurs from the age of 45-65 [3]. At monthly visits, after an overnight fasting for at least eight hours, samples (whole blood, plasma, urine, stool) were collected and sent to accredited laboratories. Comprehensive multi-omics and clinical biochemistry data were assessed. Self-administered questionnaires on, for example, health conditions and physical activity were also completed by the participants.

The clinical biochemistry and physiological data were measured monthly. On a bi-monthly basis, plasma samples were taken and omics (proteomics and metabolomics) measurements were assessed; six to seven repeated measurements per individual are available for these data. At month 13, only 20 participants could donate samples due to the start of the COVID-19 pandemic. The detailed description of IAM Frontier data is presented in Table S1. All samples have been collected in accordance with the applicable Belgian regulations regarding the use of human body material for scientific research (Belgian Law on use of human body material, 2008) and the Belgian Royal Decree on biobanks (*Het Koninklijk Besluit betreffende de biobanken. Belgisch Staatsblad 05.02.2018. Brussels (2018)*).

Table S1: Overview of the IAM Frontier longitudinal cohort data.

Data	Measurement technique	Timepoints	Type of biological sample	Total samples
Clinical biochemistry	Clinical biochemistry, blood cell counts	Monthly	Whole blood, serum PB, fasting urine	380
Physiological data	Health examination	Monthly	-	380
Health condition, physical activities	Intake and monthly questionnaires	Monthly	-	380
Metabolomics	NMR - MS	Bi-monthly	Plasma	200
Proteomics	PEA	Bi-monthly	Plasma	200

2 IRIS pipeline for data quality check

For each selected biomarker, we perform a data quality check for ensuring that the subject observations per time series are in a stable state; if the aim is to generate IRIs in healthy individuals, this means the subjects are in a healthy condition over a period of time. The pipeline includes three steps of data quality check: 1) outliers exploration, 2) monotonic trend analysis, and 3) variance checking. The outlier's exploration checks if there are outlying observations in each time series observations of one subject. Outliers are defined in terms of the median absolute deviation (MAD). For each subject time series, the MAD threshold is computed. In particular, $\tilde{y} \pm z_{0.99} MAD$ is used to set the lower and the upper thresholds for identifying potential outliers, where $\tilde{y}$ is the median of each subject observations per time series and $z_{0.99}$ is the 99th percentile of a standard normal distribution. The MAD threshold is chosen for its robustness in describing data with a relatively small time series, as often observed in routine laboratory analyses. The 99th standard normal percentile is selected as a strict threshold that could define possible outlying observations. We also compute the thresholds and define outliers for all other features in the data (besides the selected biomarkers). The latter is necessary for assessing the possibility that an individual's test results at one time point could deviate from the healthy status, reflecting an abnormality shown by the outliers identified in several features. Thus, for each time point in every subject, the proportion is taken; the number of features with outliers proportional to the total number of features. By default, all outliers would still be included in the IRI estimation as they might contain important information and professionals' opinions are required to do otherwise. However, for this study, an arbitrary threshold of 20% is set. This threshold means if a particular measurement in the time series observations is also outlying in more than 20% of the total features, we assume abnormalities, and hence that measurement will be excluded. All outliers that are still included will be flagged in the final IRI estimates. A stable state of subject observations per time series is then assessed using the nonparametric Mann-Kendall (MK) test of monotonic trends. We argue that if a monotonic trend is present

within one each time series observations, it may suggest a decline or a progression towards a particular health condition (disease), hence an unstable state. We choose a nonparametric test, because in realistic datasets each subject contributes only short time series (i.e., small sample size). Apart from the MK test for analysing if either a monotonic increasing or decreasing trend is present, for each subject, we also compute Spearman rank correlation coefficients between subject observation per time series and the time point covariate. In case a monotonic trend and the correlation are statistically significant, the subject is excluded from the IRI estimation. The last step in the data quality check involves the computation of the variance in each time series observations. To support the argument that the subject should be in a stable state, the variance of each time series observations should also remain small for all subjects. A similar MAD threshold is computed; subjects with variance exceeding the threshold are excluded. The pipeline overview can be consulted in Figure S1.

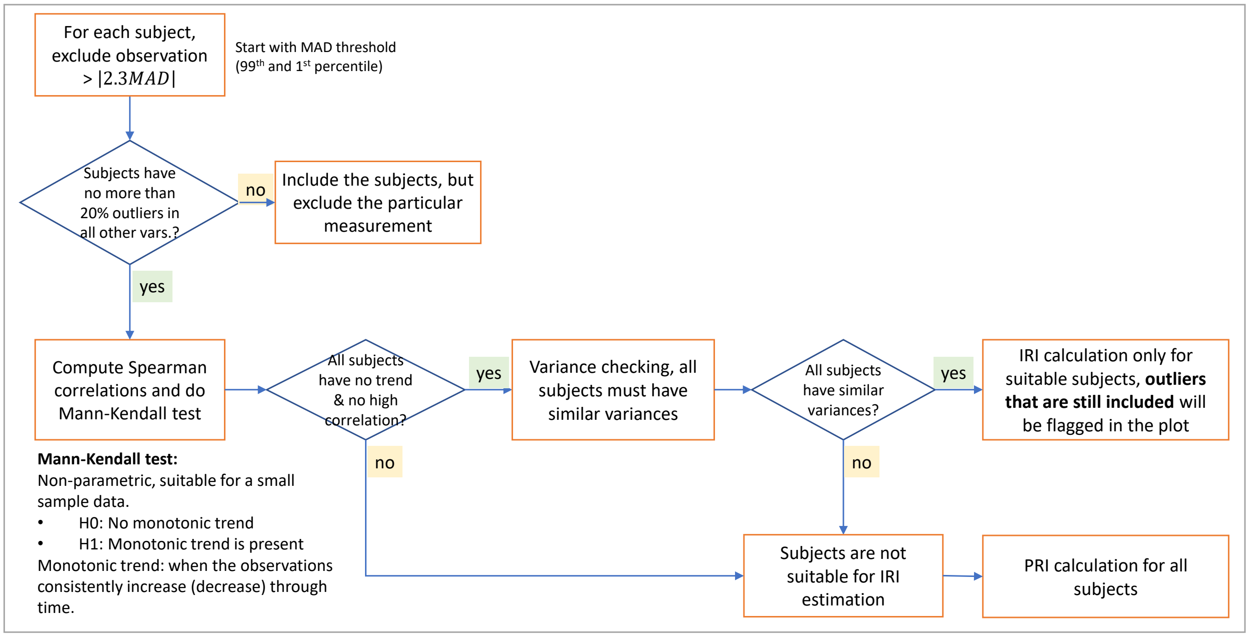

Figure S1: IRI pipeline implemented in the study.

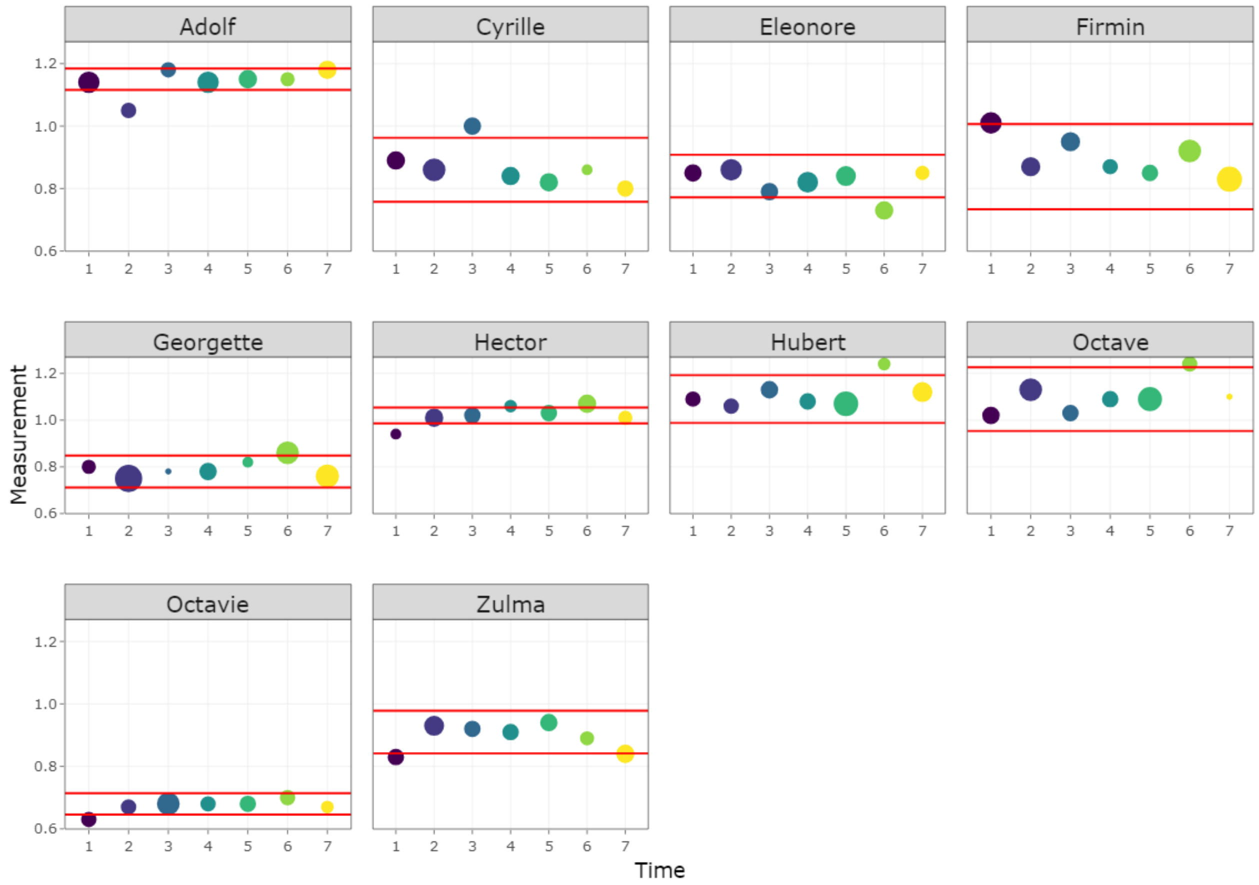

Figure S2: Outlier analysis step of creatinine in the IAF clinical biochemistry data. Outlying observations are shown as the circles outside the MAD thresholds (red horizontal lines).

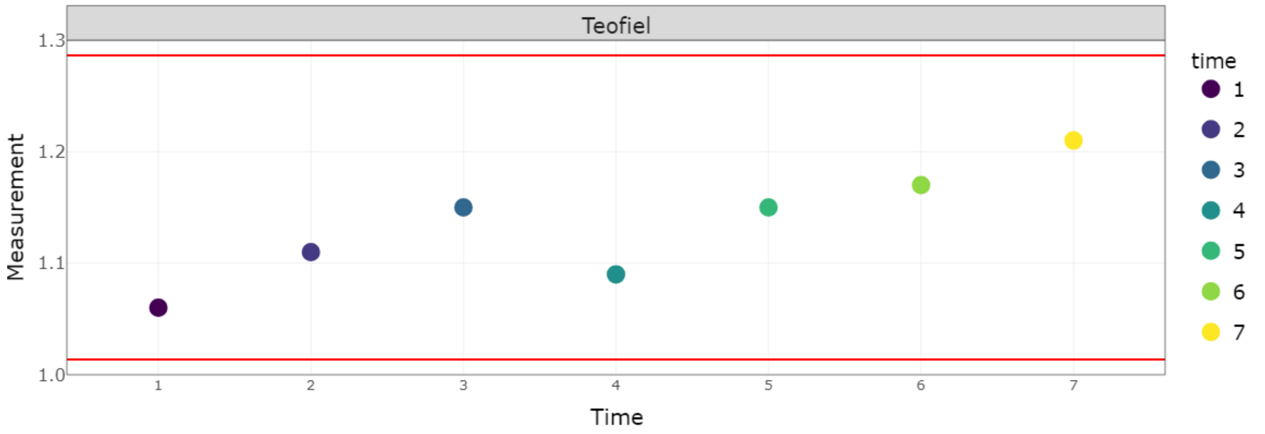

Figure S3: Trend analysis of creatinine in the IAF clinical biochemistry data. This subject has a significant monotonic trend and a high Spearman correlation coefficient ($r\hat{h}o = 0.8649$. This subject will be excluded from the IRI estimation.

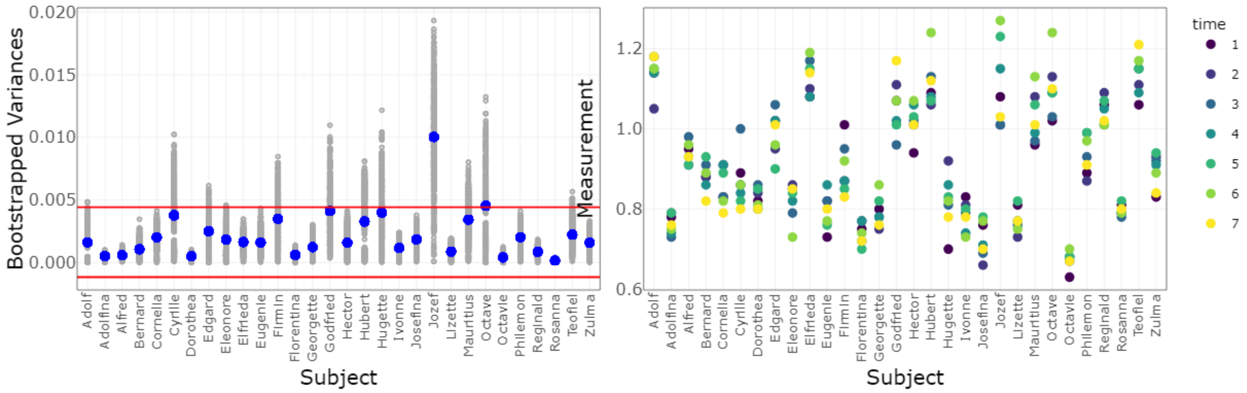

Figure S4: Variance checking of creatinine in the IAF clinical biochemistry data. Two subjects (Jozef and Octave) have variances outside the MAD thresholds and they will be excluded from the IRI estimation.

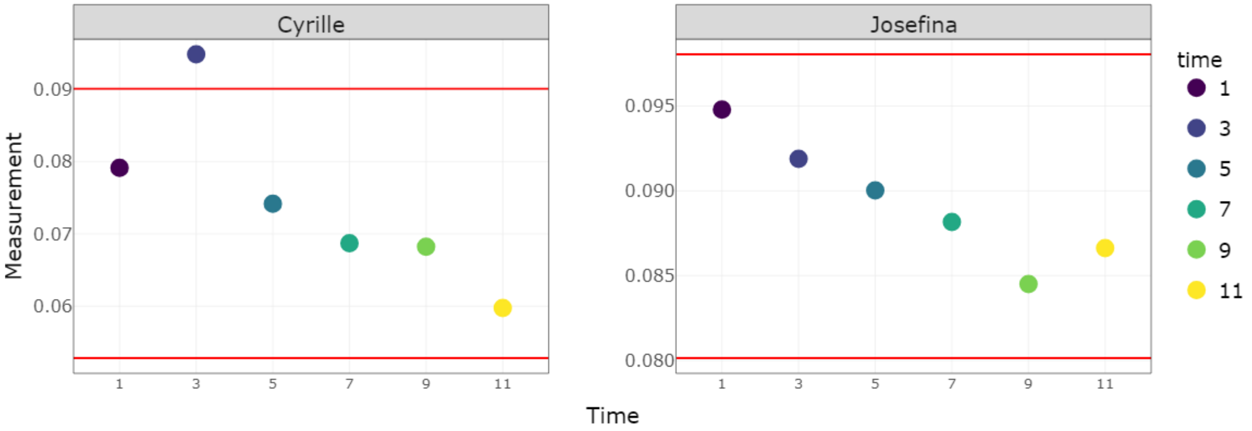

Figure S5: Trend analysis of citrate in the IAF metabolomics data. Two subjects have significant monotonic trends and high Spearman correlation coefficients ($\hat{\rho}_{Cyrille} = -1$ and $\hat{\rho}_{Josefina} = -0.9429$). These subjects will be excluded from the IRI estimation.

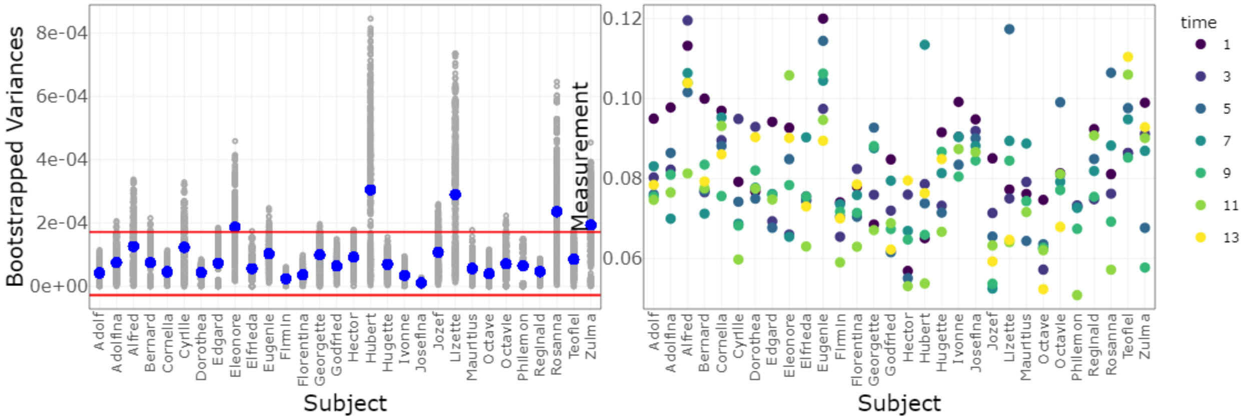

Figure S6: Variance checking of citrate in the IAF metabolomics data. Five subjects have variances outside the MAD thresholds and they will be excluded from the IRI estimation.

3 Variable Selection for Omics Data

3.1 Metabolomics and proteomics in the IAF data

The 10-year cardiovascular disease (CVD) risk scores are computed for all IAF participants, using the clinical biochemistry and physiological data, with the atherosclerosis cardiovascular diseases (ASCVD) calculator (see Appendix 7 in GoffJr et al. [2]). Variables included the risk score calculation are: age, sex, race, smoking status (yes/no), systolic blood pressure, diabetes (yes/no), HDL cholesterol, total cholesterol, and treatment for hypertension (yes/no). From the results, the male participants have 1.1-35.4% risk of CVD event(s) in the next 10 year, whereas the risk is only 0.3-3.9% in females.

These risk scores are calculated for each individual at each time point, and later they are utilised in the variable selection step of the data processing workflow. We associate both metabolomics and proteomics datasets (in two separate models) with the estimated CVD risk scores. We use the simultaneous penalised linear mixed models (SP-LMM) implemented in *splmm* R package for performing a simultaneous variable selection of both fixed and random effects using a class of penalty functions as explained in Yang and Wu [5]. This method was particularly developed for the purpose of variable selection in high-dimensional data. The following model is fitted for each metabolomics and proteomics data,

$$\mathbf{RS}_i = \alpha_0^{(n)} + \alpha_1^{(n)}\text{age}_{ij} + \alpha_2^{(n)}\text{sex}_i + \sum_{k=1}^{p} \alpha_{k+2}\mathbf{X}_{ki} + a_0 + a_1^{(n)}\text{age}_{ij} + a_2\text{sex}_i + \epsilon_i, \quad (1)$$

where for the i-th individual with n_i repeated measurements, $\mathbf{RS}_i$ is the matrix of estimated ASCVD risk scores, $\mathbf{RS}_i = (RS_{i1}, RS_{i2}, \ldots, RS_{in_i})^T$ and $\mathbf{X}_{ki}$ is the matrix of fixed effects of the k-th protein/metabolite covariates, $\mathbf{X}_{ki} = (X_{ki1}, X_{ki2}, \ldots, X_{kin_i})^T$. Fixed and random effect coefficients are denoted by $\alpha_k, k = 1, 2, \ldots, p$, and (a_0, a_1, a_2), respectively. The symbol (n) indicates that for these parameters we keep them unpenalised. Two penalisation techniques are considered: LASSO and Smoothy Clipped Absolute Deviation (SCAD). The best model with the lowest Bayesian Information Criterion (BIC) is selected.

3.2 Metabolomics in the IBS data

The variable selection procedure is also applied for the NMR metabolomics measurements in the IBS data. Generalised linear mixed models with ℓ_1 penalisation are fitted to the log10-transformed metabolomics measurements and a binary response of IBS status. The following model is considered:

$$\text{logit}(\pi(x)) = \ln\left(\frac{\pi(x)}{1-\pi(x)}\right) = \alpha_0 + \alpha_1\text{age}_{ij} + \alpha_2\text{sex}_i + \sum_{k=1}^{p} \alpha_{k+2}\mathbf{X}_{ki} + a_0 + a_1\text{age}_{ij}, \quad (2)$$

where $\pi(x)$ is the probability of an individual is diagnosed with IBS, given the linear combination of individual's age, sex, and the set of metabolites. We perform a penalisation procedure implemented in the *glmmLasso* R package for estimating the effect sizes and selecting the most discriminating variables. Again, the best model with the lowest BIC is selected.

3.3 Results of Variable Selections

For the variable selection of the IAF metabolomics and proteomics data, we identified five and ten most discriminating metabolites and proteins related to the 10-year CVD risk scores. Table S2 explains the list of selected proteins and metabolites together with the estimated effect size. Citrate and phospholipids in small density LDL cholesterol (S-LDL-PL) from the metabolomics dataset give the largest effect size. Analogously, ICAM-2, SELP, and KIM1 give the largest effect size from the proteomics dataset. The scatter plots of these biomarkers against the CVD risk scores in Figure S7A shows a negative association between the CVD risk scores and citrate as well as the ICAM-2 protein, consistent with their negative estimated effect sizes. We performed a data quality check as explained in the IRIS pipeline for each metabolite and protein. Figure S7B presents the outlier identification of citrate in four subjects. For this metabolite, we found that Octave's citrate measurement at the first time point is outlying, and at the same time point, his measurements are also outlying in 53 out of 249 metabolites (21%), represented by the size of the circle. Therefore, we excluded his first citrate measurement from the data. The second measurement of Cyrille should also be removed (67 out of 249 or 27% of metabolites are outlying at this time point), but at the end, we completely excluded Cyrille from the estimation as a monotonic trend is significantly present. Based on the trend analysis and variance checking (see Figure S5-S6), seven subjects were eventually excluded.

Table S2: Estimated effect sizes of each metabolites and proteins from the IAF metabolomics and IAF proteomics association analysis against CVD risk scores, and from the IBS metabolomics association analysis against the IBS status. The selected biomarkers are in bold face.

IAF Metabolomics		IAF Proteomics		IBS Metabolomics		
Biomarker	Estimate	Biomarker	Estimate	Biomarker	Estimate	Odds ratio
(Intercept)	-24.1155	(Intercept)	-28.0100	(Intercept)	0.1802	1.1974
Age	0.3913	Age	0.0266	Age	0.0137	1.0138
sex	5.7284	sex	1.2833	**b_arabinose**	**1.0618**	**2.8915**
**Citrate**	**-5.2821**	CCL28	3.0866	**Glucose**	**0.3398**	**1.4046**
Glucose	0.3252	GRN	1.6412	**Hypoxanthine**	**-3.0712**	**0.0464**
Linoleic acid	0.5356	**ICAM-2**	**-11.8859**			
Lactate	0.2132	IL2-RA	3.3319			
**S_LDL_PL**	**20.2414**	**KIM1**	**3.3424**			
		MARCO	0.2771			
		MMP-10	-0.7601			
		**SELP**	**6.0702**			
		SHPS-1	-2.8197			
		THPO	-0.2302			

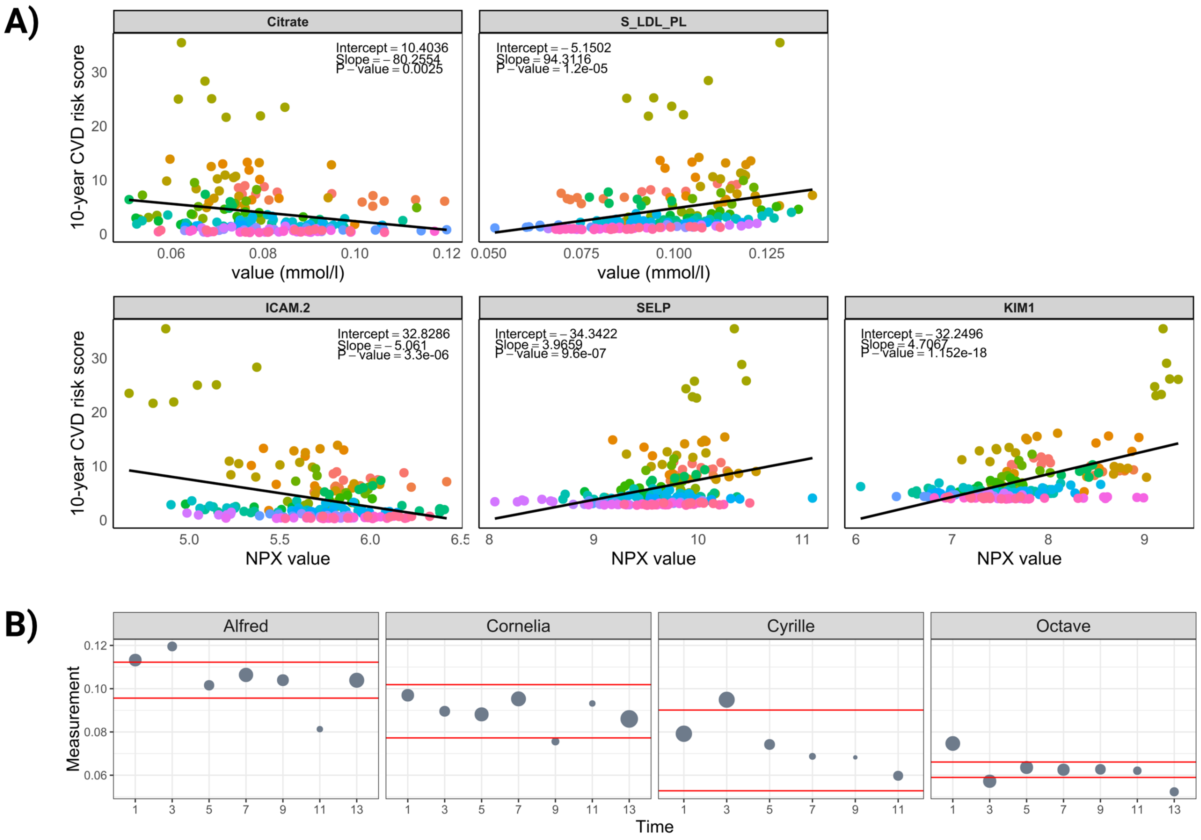

Figure S7: **(A)** Scatter plots of the selected biomarkers in the metabolomics and proteomics datasets with their corresponding regression lines (black solid lines). Each dot refers to a measurement at one time point; different colors are assigned to different subjects. High citrate and ICAM-2 abundances suggest lower 10-year CVD risk scores. Parameters were estimated in 30 subjects using simple linear regression models. **(B)** Outlier analysis step for citrate in the IAF metabolomics data. Outlying observations are shown as the circles outside the MAD thresholds (red horizontal lines). The size of the circles represents the percentage of outlying observations at the same time point over all features in the dataset. An outlying observation is removed if this percentage is greater than 20% i.e. the observation is also outlying in more than 20% of the features. An example: Cyrille's measurement at the second time point.

4 IRI performance in creatinine

We evaluated the IRI performance of creatinine by computing the IRI coverage in different number of observations per subject and different covariate models, using the same data with 27 subjects remaining after the data quality check. See Figure S8. The IRI coverage refers to the probability that an interval covers a new test result for example, the creatinine test result at the next time point. A good method gives a coverage close to the nominal level (e.g., 95%). As shown in the figure, we found that when the observations per time series $n \geq 7$, the coverage became stable around 95% for the models with only sex covariate, with both age and sex covariates, and without any covariates. Moreover, at n=7, models with both age and sex covariates and the model without any covariates gave the best coverage. We can also interpret this result in two ways: 1) the IRI model should not include age alone, but rather in combination with sex, to produce good coverages, and 2) at least seven observations per time series are needed to obtain IRIs with stable coverages close to 95%. Note that this conclusion is not to be considered as a general guideline for creatinine for future use; here we have used only a small dataset for the evaluation. When more data are available, we recommend performing a new, large-scale evaluation.

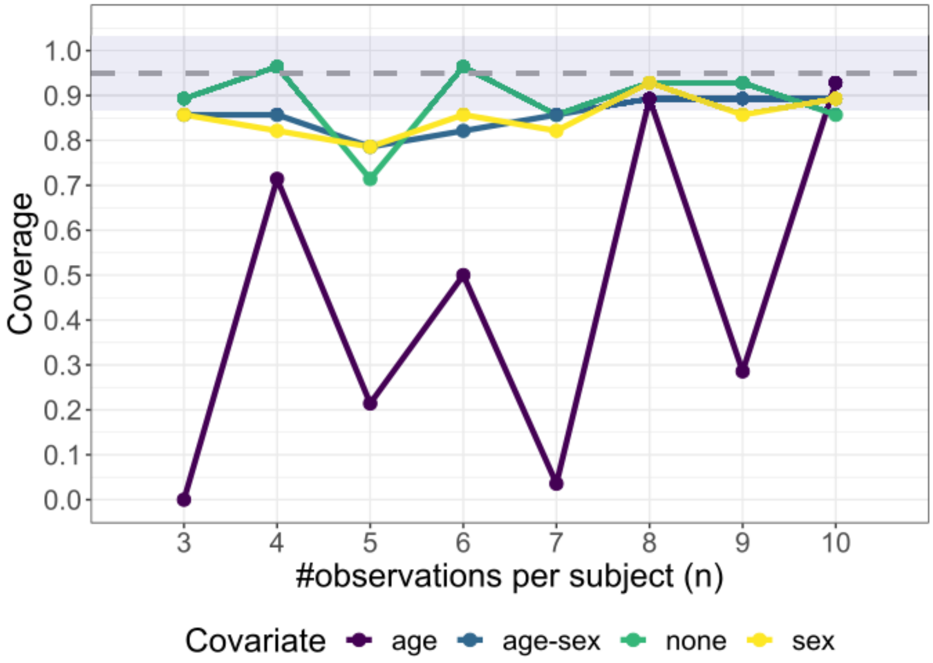

Figure S8: Coverage of IRIs (± std. deviation in the blue area) in IAF creatinine with different observations per time series fitted in different models. Model with only age as covariate gives unstable coverages. In the model with age and sex, only sex, and without covariates, at least seven observations per time series are needed to produce IRIs with coverage close to 95%. Small sample size data were used; hence a general recommendation cannot be drawn.

5 IRI estimates in omics

5.1 Metabolomics and proteomics in the IAF data

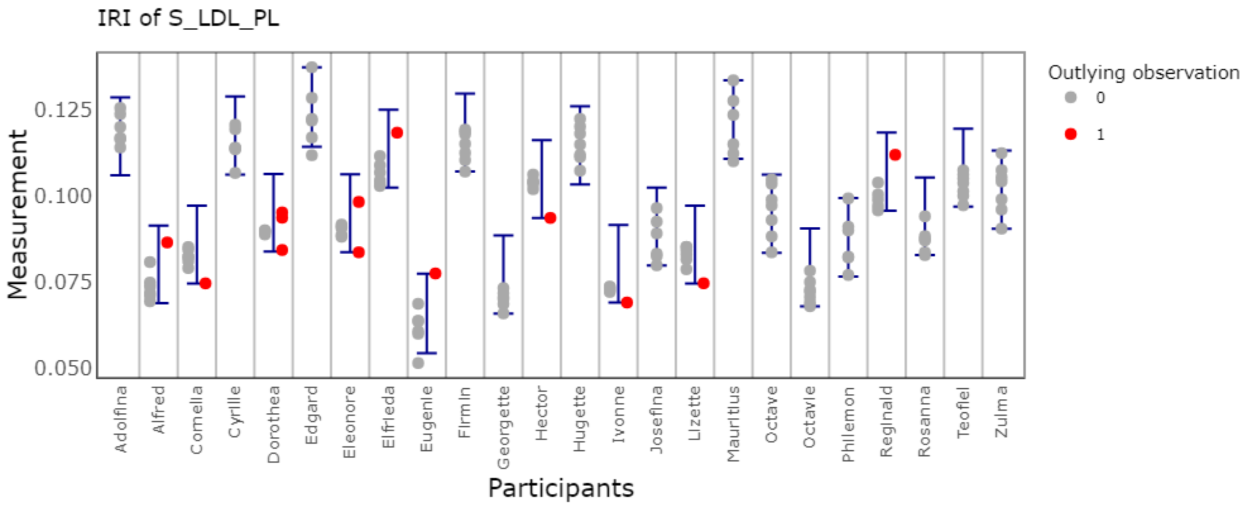

Figure S9: IRI of S-LDL-PL

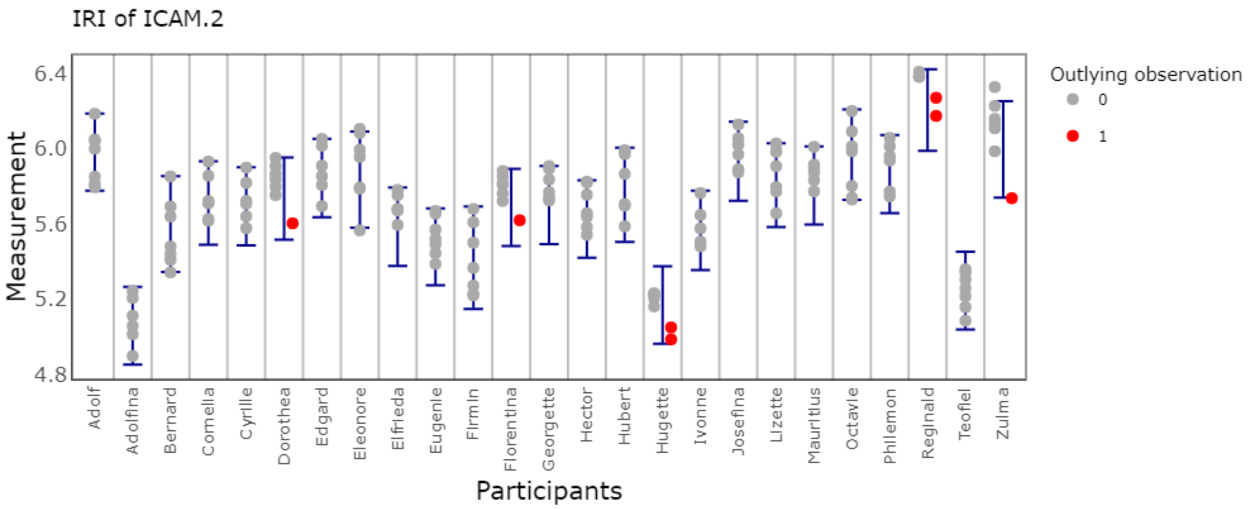

Figure S10: IRI of ICAM-2

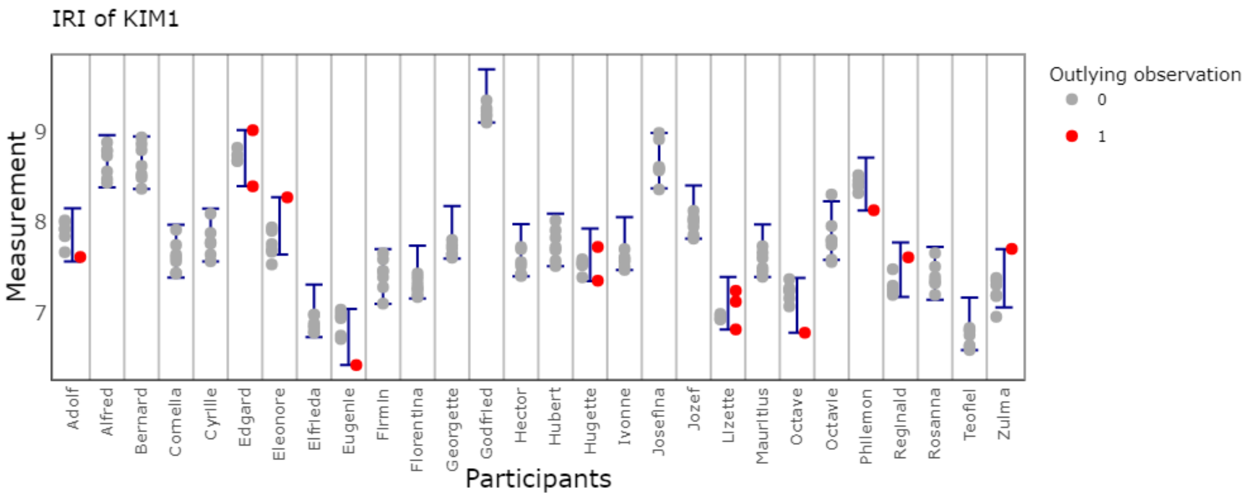

Figure S11: IRI of KIM-1

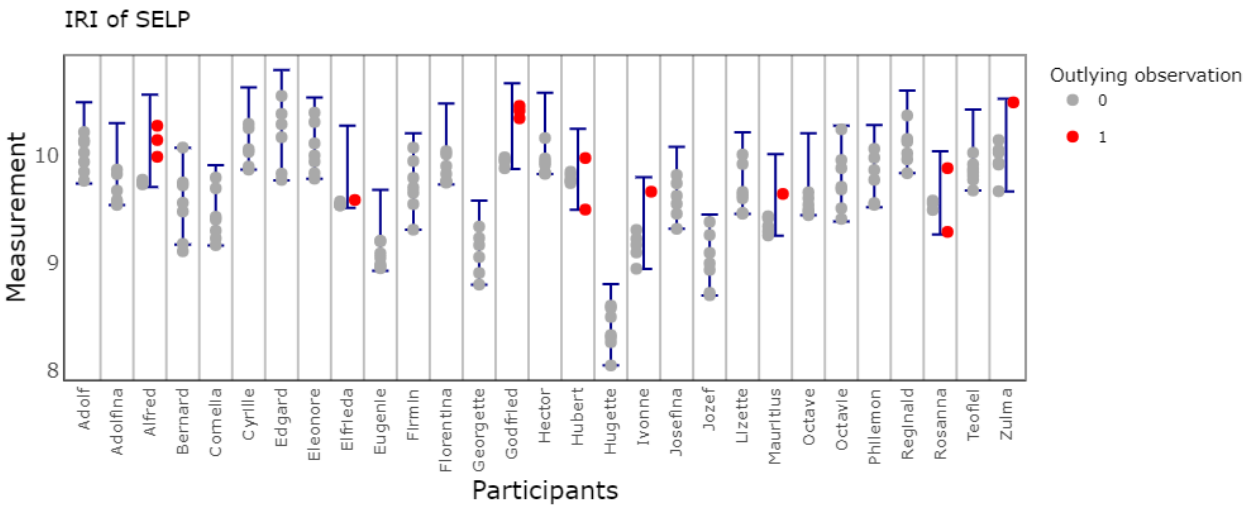

Figure S12: IRI of SELP

5.2 Metabolomics in the IBS data

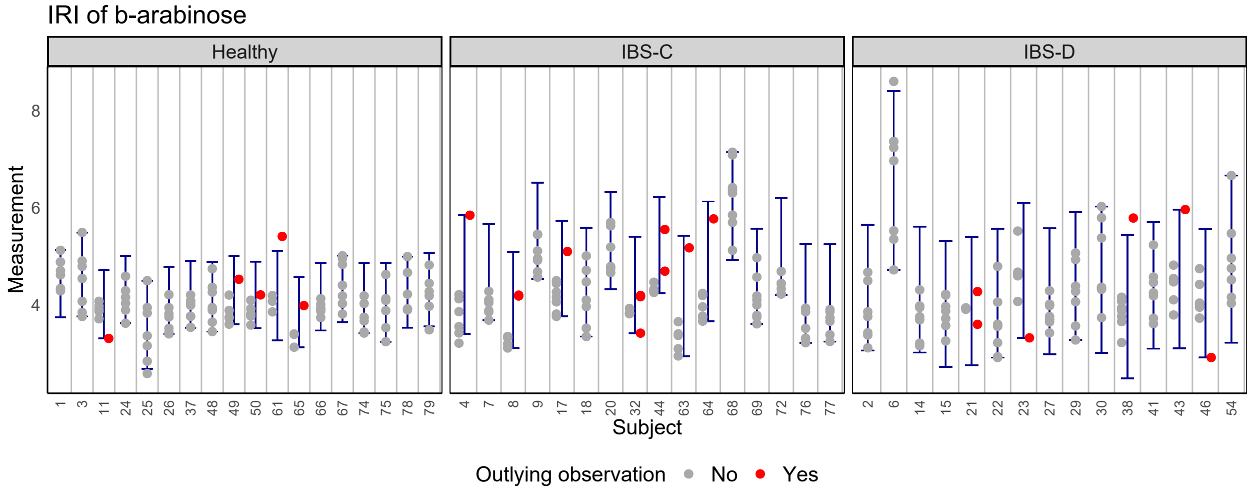

Figure S13: IRI of b-arabinose

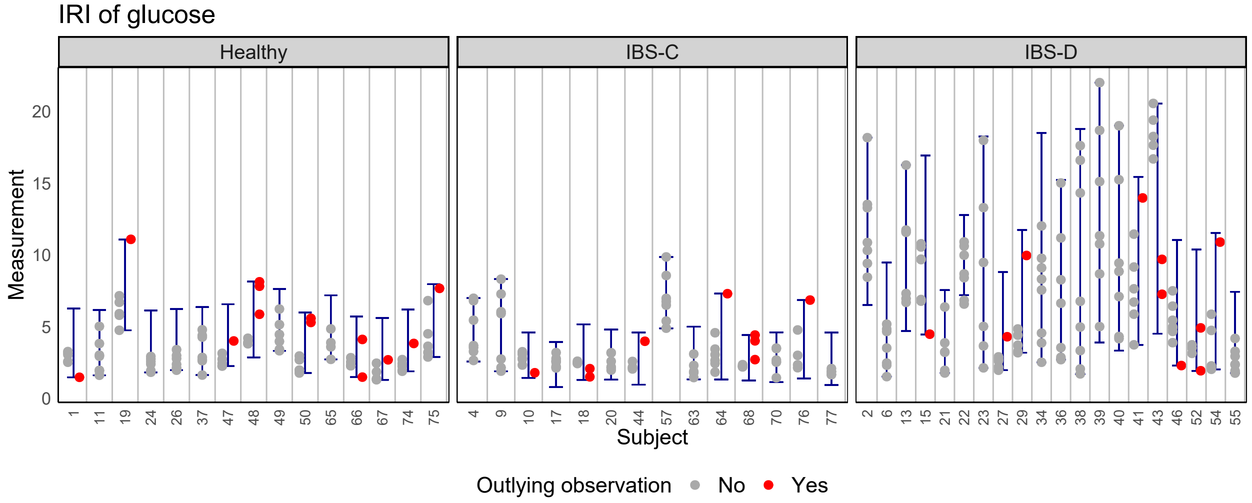

Figure S14: IRI of glucose

Table S3: P-value of the standard student t-test used for comparing the mean of IRI widths between healthy individuals and IBS patients, after the Benjamini-Hochberg correction. The bold font face indicates when the two means are statistically significant at 5% significance level. Metabolites with significant P-values in all three pairs are also made italic.

Metabolites	Healthy vs IBS-C	Healthy vs IBS-D	IBS-C vs IBS-D
Deoxycholic acid	**0.0455**	0.0568	0.5696
Cholic acid	**0.0010**	**0.0016**	**0.0007**
Ursodeoxycholic acid	0.0612	0.5591	0.0628
Lithocholic acid	0.1373	**0.0002**	**0.0357**
Chenodeoxycholic acid	0.8482	**0.0008**	**0.0009**
X2 Methylbutyrate	**0.0117**	0.8693	**0.0477**
Lactate	**0.0004**	**0.0098**	**0.0000**
Alanine	0.8482	**0.0058**	**0.0301**
Tyrosine	0.1393	**0.0005**	**0.0127**
Isoleucine	**0.0455**	**0.0002**	0.1913
Leucine	0.7971	0.1024	**0.0357**
Valine	**0.0416**	**0.0000**	**0.0026**
Lysine	0.0719	**0.0130**	0.2855
Succinate	**0.0455**	0.0627	**0.0309**
Glycine	0.8482	**0.0008**	**0.0007**
b-arabinose	**0.0000**	**0.0000**	**0.0000**
b-xylose	**0.0033**	**0.0000**	**0.0000**
Acetate	0.8457	0.0657	**0.0408**
Propionate	**0.0455**	0.8233	0.1927
Butyrate	0.3211	0.2341	**0.0372**
Glucose	**0.0013**	**0.0000**	**0.0000**
Isovalerate	0.8482	0.0752	0.0939
Uracil	0.8482	0.2488	0.3110
Hypoxanthine	**0.0000**	**0.0000**	**0.0000**

6 IRI ranges in IAF data

Two methods are involved in the estimation of personalised version of reference intervals, 1) Personalised Reference Intervals (PrRI), parametric approach [1], and 2) Individual Reference Intervals (IRI) via joint quantile models, non-parametric approach [4]. In both methods, a data quality check is performed before calculating the individual reference intervals. This step involves checking for trends in each time series and identifying any outlying observations. Ten clinical biochemistry variables are selected for this implementation. For each individual in the dataset, we computed their IRI estimates and calculated the interval lengths (the difference between the upper and lower bounds), or we later call it the interval range. Interval ranges are significant because they affect how test results are interpreted. If an IRI has a wide interval range, deviations from 'normal' may go undetected, leading to false negatives, and conversely, a narrow range may result in false positives. Table S3 presents the mean and median of the interval ranges for each of the clinical biochemistry variables.

Table S4: Mean and median IRI ranges for ten clinical biochemistry variables.

**Clinical biomarkers**	**Method**	**Mean_range**	**Median_range**
Total bilirubine_mg/dL	IRI	0.6008	0.5552
Total bilirubine_mg/dL	PrRI	0.7182	0.6229
Calcium_mmol/L	IRI	0.2350	0.2241
Calcium_mmol/L_serum PB	PrRI	0.2584	0.2446
Creatinin_mg/dL	IRI	0.2146	0.2089
Creatinin_mg/dL	PrRI	0.2234	0.2117
Glucose_mg/dL	IRI	17.3035	16.3876
Glucose_mg/dL	PrRI	20.1157	17.8094
HDL-cholesterol_mg/dL	IRI	19.7584	17.4747
HDL-cholesterol_mg/dL	PrRI	21.4861	20.1872
HbA1c_mmol/mol	IRI	4.0741	4.0766
HbA1c_mmol/mol	PrRI	4.7610	4.7011
Haemoglobin_g/dL	IRI	1.9955	1.9575
Haemoglobin_g/dL	PrRI	2.4572	2.4124
Magnesium_mmol/L	IRI	0.1359	0.1322
Magnesium_mmol/L	PrRI	0.1460	0.1450
TSH_mU/L	IRI	1.1262	0.9660
TSH_mU/L	PrRI	1.6413	1.4631
Uric acid_mg/dL	IRI	1.6262	1.6054
Uric acid_mg/dL	PrRI	1.7555	1.6982

Figure S15 shows the distribution of interval ranges for each clinical biochemistry variable across the two methods. Notably, the median range is generally lower in the IRI method, which also exhibits less variability. This outcome can be attributed to an assumption built into the IRI algorithm: although the reference intervals are personalized, both the location

and range of these intervals are constrained by the overall population variance. This assumption is reasonable, as the individuals in the dataset are likely from the same population and share similar biological characteristics.

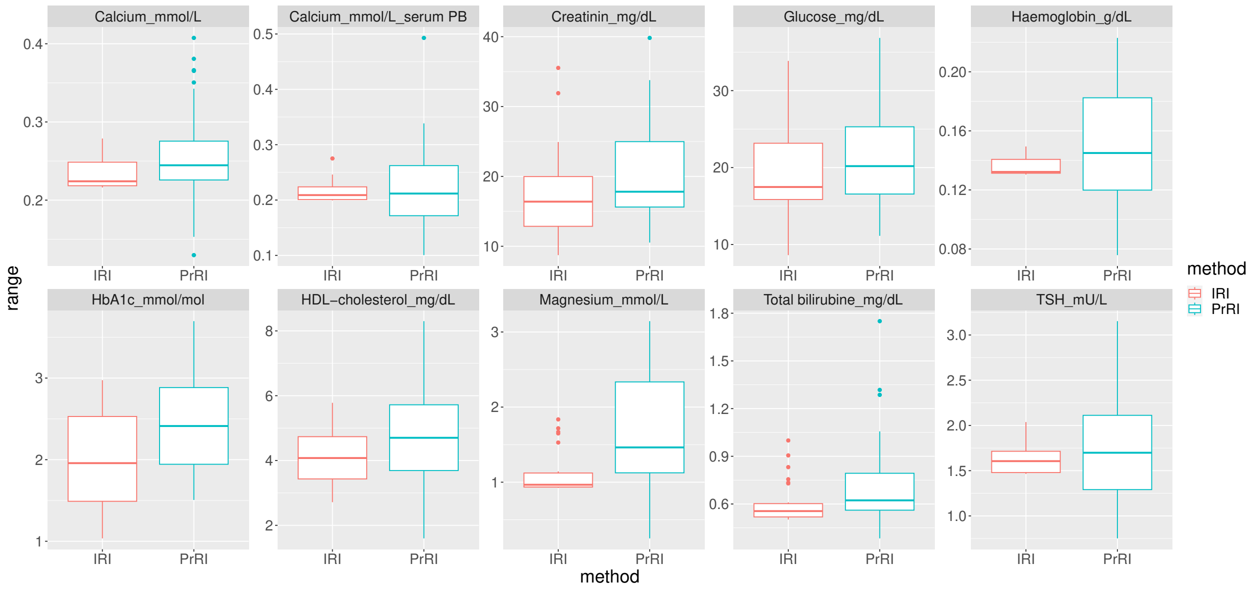

Figure S15: Distribution of the IRI ranges in ten clinical biochemistry variables.

7 MIMIC-III data processing and IRI estimation

7.1 Data extraction

Clinical laboratory measurements were extracted from the `labevents` table and linked to ICU stays using identifiers at the subject-level (`subject_id`), hospital admission (`hadm_id`), and ICU stay (`icustay_id`). Laboratory measurements were filtered to include only numeric results (`valuenum`) with valid timestamps (`charttime`). For the analyses presented in this study, serum creatinine was selected as a representative biomarker due to its frequent measurement in ICU settings and its relevance to circulatory and renal function.

Information on vasopressor administration was extracted from the `inputevents_cv` and `inputevents_mv` tables. Vasopressor start time was defined as the first recorded administration of a vasopressor agent during an ICU stay. This time point was used as a clinically meaningful and precisely time-stamped marker of acute physiological deterioration.

Demographic variables, including age and sex, were obtained from the `patients` and `admissions` tables and linked at the subject level.

7.2 Cohort selection

ICU stays were eligible for inclusion if they satisfied the following criteria:

- At least one documented vasopressor initiation during the ICU stay.
- Availability of longitudinal creatinine measurements before and after vasopressor initiation.
- A minimum number of observations within the predefined baseline window (see Section S4).

Each ICU stay was treated as an independent longitudinal unit of analysis. Subjects with multiple ICU stays could therefore contribute more than one time series, provided that each ICU stay satisfied the inclusion criteria.

7.3 Time window definition

All laboratory measurements were temporally aligned relative to the vasopressor initiation time, which was defined as time zero. Four non-overlapping time windows were defined:

- **Baseline window:** -72 to -24 hours, used for IRI estimation.
- **Buffer window:** -24 to -12 hours, excluded to avoid contamination by early transitional changes.
- **Prediction window:** -12 to 0 hours, used for early-warning evaluation.
- **Post-event window:** 0 to $+24$ hours, used for confirmatory analyses only.

Only data from the baseline window were used for model fitting. Prediction, buffer, and post-event windows were strictly excluded from IRI estimation.

7.4 Stability screening and minimum observation requirement

To ensure valid estimation of individual reference intervals, baseline data were required to satisfy stability assumptions. Specifically:

- A minimum of five observations within the baseline window was required. This threshold reflects a pragmatic compromise between ensuring sufficient longitudinal information for IRI estimation and retaining an adequate number of eligible ICU stays for analysis. More stringent requirements (e.g., seven or more observations) resulted in a substantial reduction in sample size and were therefore not adopted in this study.
- Monotonic trends were assessed using the Mann–Kendall trend test.
- Extreme variance inflation was evaluated using within-subject variance criteria.

ICU stays failing these checks were excluded from IRI estimation. These criteria serve as methodological quality control measures rather than indicators of clinical irrelevance.

7.5 IRI and PrRI estimation

Individual reference intervals (IRI) were estimated using the PJQM2 framework [4]. The model jointly estimates subject-specific lower and upper quantiles while borrowing information across individuals. Age and sex were included as fixed-effect covariates. For comparative purposes, personalised reference intervals (PrRI) were also estimated using the PrRI method proposed by Coskun *et al.* [1]. Both IRI and PrRI were computed using the baseline data only that satisfied the stability assumptions. Performance comparisons between IRI and PrRI were conducted using prediction-window data, focusing on early-warning metrics such as lead time, sensitivity, and discriminative ability.

Evaluation metrics

Early-warning performance was assessed using the following metrics:

- **Specificity:** proportion of ICU stays within the baseline window. This specificity baseline was interpreted as a calibration property rather than a discriminative metric, given that the IRIs are constructed to achieve nominal coverage within the estimation data.
- **Lead time:** time between the first detected deviation and vasopressor initiation.
- **Sensitivity:** proportion of ICU stays with at least one detected deviation in the prediction window.
- **AUROC:** computed using continuous deviation scores when defined.
- **Post-event breach rate:** proportion of post-event observations outside the interval.

8 IRIS Shinyapp demonstration

This demonstration can be found in the IRIS Shinyapp user manual: (`https://dsi-uhasselt.shinyapps.io/IRIS/`).

1. IRI concept and data requirement

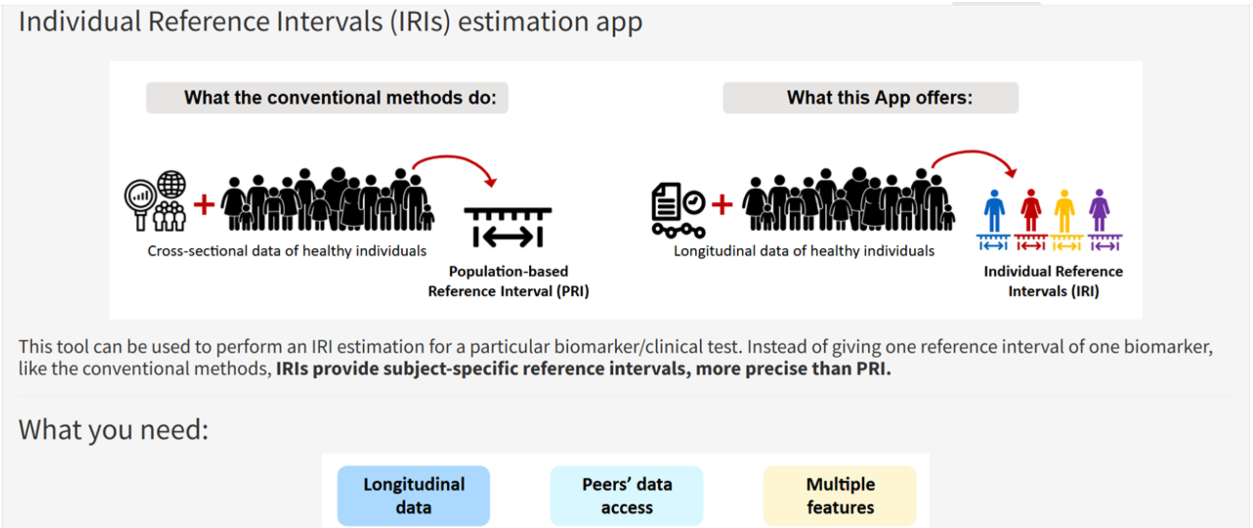

2. IRI pipeline and objectives

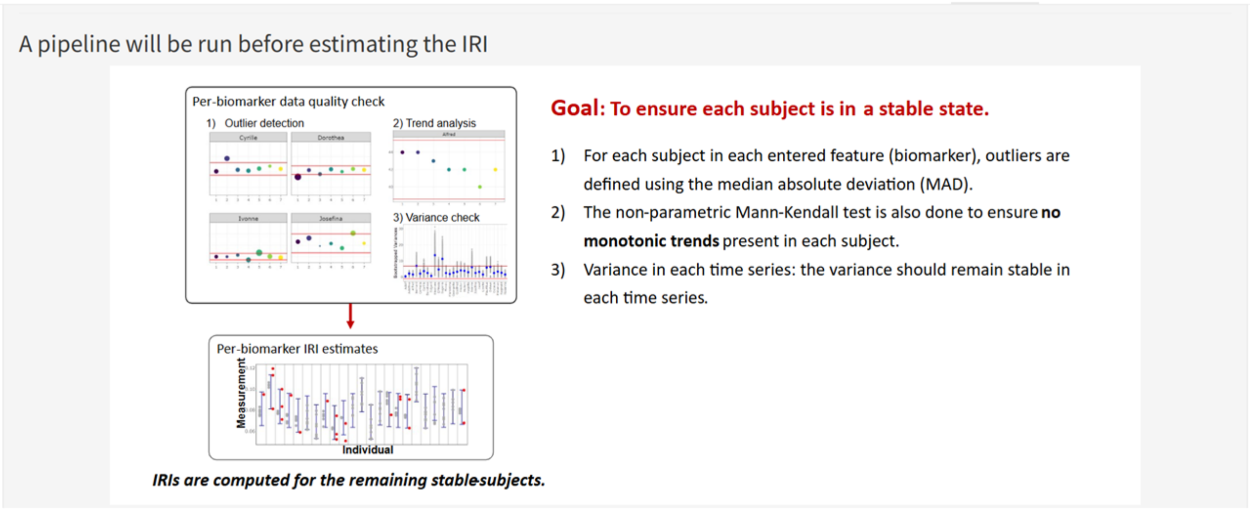

3. Demonstration in sample data

Data upload procedures:

In order to use this tool, load your data set using *Analysis > Data Upload* tab.

The data must be in a *wide format* and the first two columns should indicate the `subject` and `time` indices. As an example:

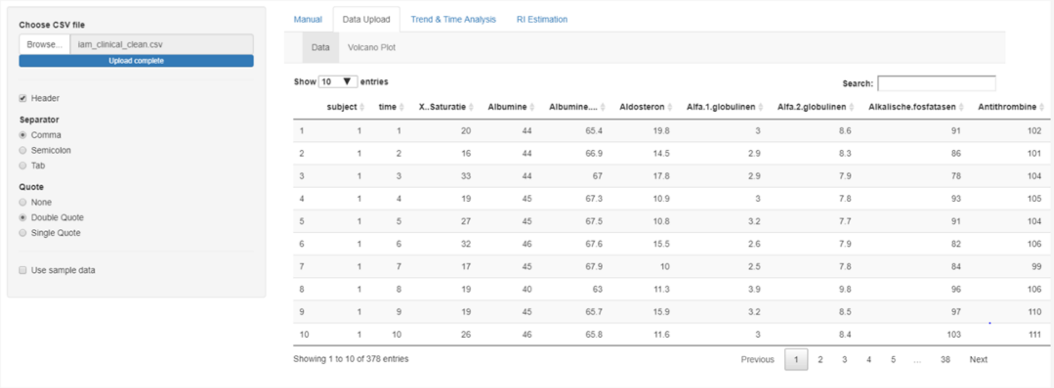

	subject	time	X..Saturatie	Albumine	Albumine....	Aldosteron	Alfa.1.globulinen	Alfa.2.globulinen	Alkalische.fosfatasen	Antithrombine
1	1	1	20	44	65.4	19.8	3	8.6	91	102
2	1	2	16	44	66.9	14.5	2.9	8.3	86	101
3	1	3	33	44	67	17.8	2.9	7.9	78	104
4	1	4	19	45	67.3	10.9	3	7.8	93	105
5	1	5	27	45	67.5	10.8	3.2	7.7	91	104
6	1	6	32	46	67.6	15.5	2.6	7.9	82	106
7	1	7	17	45	67.9	10	2.5	7.8	84	99
8	1	8	19	40	63	11.3	3.9	9.8	96	106
9	1	9	19	45	65.7	15.9	3.2	8.5	97	110
10	1	10	26	46	65.8	11.6	3	8.4	103	111

An overview of trends and correlations for all biomarkers is presented in the *Volcano Plot* tab.

You can continue the analysis by selecting a feature/biomarker in the *Trend & Time Analysis*. This feature will be carried out in the next steps until [illegible]

4. Example of estimated IRI

An example of estimated IRIs after leaving the pipeline:

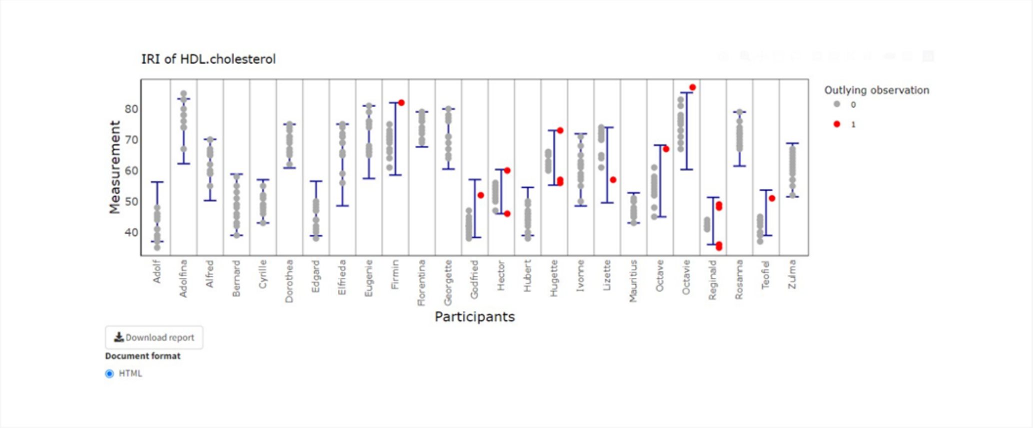

For each individual, the IRIs are indicated by the **blue error bars** . They were estimated from the previous/historical measurements indicated by the full circles. The red circles refer to outlying observations (included in the estimation).

These IRIs are designed **to interpret the new test results or the future measurements** of each subject.

The data quality check results as well as the IRI estimates can be retrieved by clicking the `Download report` button.

References

[1] A. Coşkun, S. Sandberg, I. Unsal, C. Cavusoglu, M. Serteser, M. Kilercik, and A.K. Aarsand. Personalized reference intervals in laboratory medicine: A new model based on within-subject biological variation. *Clinical Chemistry*, 67(2):374–384, 2021. doi: doi.org/10.1093/clinchem/hvaa233.

[2] D.c. GoffJr, D.M. Lloyd-Jones, and G. Bennet. 2013 acc/aha guideline on the assessment of cardiovascular risk: a report of the american college of cardiology/american heart association task force on practice guidelines. *Circulation*, 129(suppl 2):S49–S73, 2014. doi: 10.1161/01.cir.0000437741.48606.98.

[3] W. MacNee, R.A. Rabinovich, and G. Choudhury. Ageing and the border between health and disease. *European Respiratory Journal*, 44(5):1332–1352, 2014. ISSN 0903-1936. doi: 10.1183/09031936.00134014.

[4] M. Pusparum, G. Ertaylan, and O. Thas. Individual reference intervals for personalised interpretation of clinical and metabolomics measurements. *Journal of Biomedical Informatics*, 131:104111, 2022. doi: 10.1016/j.jbi.2022.104111.

[5] L. Yang and T.T. Wu. Model-based clustering of high-dimensional longitudinal data via regularization. *Biometrics*, pages 1–14, 2022. doi: 10.1111/biom.13672.
